# Self-Combustion Glycine Nitrate Process Prepared CuAlO_2_ Nanopowder Catalyst Applied into the Carbendazim Electrochemical Assessment and Computational Prediction

**DOI:** 10.1021/acs.langmuir.5c03513

**Published:** 2025-10-05

**Authors:** Sivaramakrishnan Vinothini, Chung-Lun Yu, Rohith Ramasamy, Rajendran Surya, Rajadurai Vijay Solomon, Vijayalakshmi Pandurangan, Subramanian Sakthinathan, Ching-Lung Chen, Te-Wei Chiu

**Affiliations:** † Department of Materials and Mineral Resources Engineering, 34877 National Taipei University of Technology, No. 1, Section 3, Chung-Hsiao East Road, Taipei 106, Taiwan; ‡ Institute of Materials Science and Engineering, 34877 National Taipei University of Technology, Taipei 10608, Taiwan; § Centre of Molecular Medicine and Diagnostics (COMManD), Saveetha Dental College and Hospitals, Saveetha Institute of Medical and Technical Sciences, Saveetha University, Chennai 602105, India; ∥ Department of Chemistry, Madras Christian College (Autonomous), East Tambaram, Chennai, Tamil Nadu 600 059, India; ⊥ Department of Safety, Health and Environmental Engineering, 56082 Ming Chi University of Technology, New Taipei City 243303, Taiwan; # Department of Chemistry, 93160 Tamil Nadu Open University, Saidapet, Chennai, Tamil Nadu 600 015, India

## Abstract

This work aims to develop an electrochemical sensor by modifying a glassy carbon electrode (GCE) with CuAlO_2_ nanopowder for the detection of carbendazim (CBZ). The CuAlO_2_ nanopowder, characterized by its high surface area and porous nature, was synthesized via a highly efficient glycine nitrate self-combustion process (GNP). The structural features of CuAlO_2_ and the modified electrodes were thoroughly examined using X-ray diffraction (XRD), scanning electron microscopy (SEM), transmission electron microscopy (TEM), electrochemical impedance spectroscopy (EIS), and BET surface area analysis. Furthermore, the electrochemical properties of the GCE@CuAlO_2_ electrode were investigated using cyclic voltammetry (CV), differential pulse voltammetry (DPV), and electrochemical impedance spectroscopy (EIS) techniques. The modified GCE@CuAlO_2_ electrode exhibited an enhanced sensor response toward CBZ detection, showing a significantly increased oxidation peak current along with a well-defined peak potential. It demonstrated an excellent electrocatalytic activity toward CBZ sensing, achieving a broad linear detection range (0.01–800 μM), a low detection limit (1 nM), and high sensitivity (1.44 μA μM^–1^ cm^–2^). The electrical performance of the GCE@CuAlO_2_ electrode confirmed its efficient functionality, exhibiting good stability, coherence, and long-term equilibrium. In addition, the modified electrode showed outstanding specificity, selectivity, reproducibility, repeatability, long-term stability, and anti-interference capability for CBZ detection. Furthermore, using DFT calculations, the adsorption of CBZ on the CuAlO_2_ slab has been investigated, revealing exceptional sensing performance with an adsorption energy of −5.973 eV and strong interaction between CuAlO_2_ and CBZ, as confirmed by the NCI scatter plot. In conclusion, the GCE@CuAlO_2_ electrode developed via a cost-effective and straightforward method presents a promising platform for constructing highly efficient electrochemical sensors for CBZ detection at the nanomolar level in various vegetables, fruits, and different water media.

## Graphical abstract

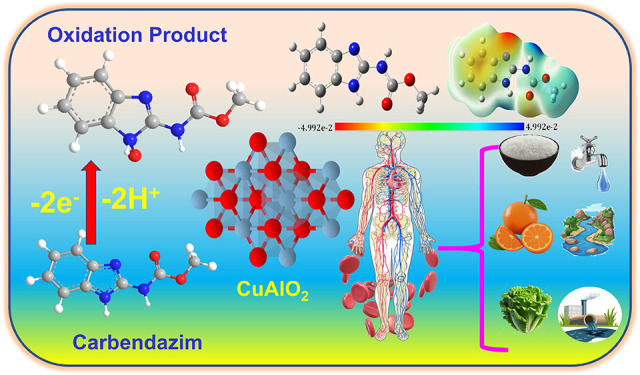

## 1 Introduction

Pesticides are widely used in agriculture for pest and weed control, as well as for enhancing crop yield, due to their cost-effectiveness and broad-spectrum efficacy. They are widely used to protect fruits and vegetables from various pathogenic threats.^1,2^ Among the various types of pesticides, benzimidazole fungicides and organophosphorus pesticides (OPs) account for nearly 70% of total pesticide usage, due to their significant contribution to productivity, quality, and economic viability.^3^ However, widespread use of these pesticides leads to residual contamination in food and the environment, including water and soil, posing significant risks to both human health and ecosystems. Pesticide residues can have adverse impacts on aquatic systems, compromise food safety, and disrupt ecological balance and human well-being.^4,5^

Carbendazim (CBZ), a benzimidazole fungicide (methyl benzimidazol-2-ylcarbamate), is among the most commonly applied pesticides, especially on citrus crops. It is typically sprayed directly onto plant foliage, either alone or in combination with other formulations.^6^ The Brazilian National Surveillance Agency (BNSA) classifies CBZ as a toxicological class III fungicide, meaning it is moderately hazardous. It is broadly used on a variety of crops, including citrus, apples, beans, soybeans, wheat, rice, maize, and cotton. CBZ is a white crystalline solid that is water-insoluble and is extensively used before and after harvest to combat fungal diseases such as mildew, mold, rot, spot, and scorch.^7,8^ Furthermore, CBZ is used to protect various materials, including polymers, fibers, leather, rubber, and films, from fungal degradation.^9,10^ It is also used as a preservative to extend the shelf life of fruits.^11^

Due to its potential toxicity, the European Union has set a maximum residue limit (MRL) of 0.1 mg/kg for CBZ. The World Health Organization classifies it as a hazardous chemical with possible carcinogenic effects in humans. As a result of its toxicity and persistence, CBZ has been banned in countries such as the United States, Australia, and much of the European Union.^12,13^ However, its production and application continue in countries like the UK, Portugal, and developing nations, including Brazil, China, Thailand, and India. Consequently, many nations have enforced strict MRL regulations for CBZ in food products.^14,15^ According to the Brazilian Health Regulatory Agency (ANVISA), CBZ is considered a moderate-risk pesticide with a toxicological class III classification, and the established MRL for citrus fruits is 2.62 × 10^–5^ mol/L.^16^ Overall, MRL values for CBZ in raw foods range from 0.05 to 15.0 mg/kg, depending on the type of produce, with values of 5.0 mg/kg for apples and oranges, and 1.0 mg/kg for mushrooms. The International Codex Alimentarius Commission (ICAC) has established 46 MRLs for CBZ across different crops and fruits, ranging from 0.05 to 20 mg/kg.^17,18^ The Chinese Ministry of Agriculture (MOA) has conducted risk assessments for CBZ, citing its frequent usage and environmental persistence as major concerns. Improper handling of CBZ results in its residues entering food chains and the environment, potentially reducing mammalian fertility by impairing sperm function. Due to the high chemical stability of CBZ, particularly its benzimidazole ring, degradation is slow, posing environmental and health risks as a potential human carcinogen. Its half-life exceeds 25 months under anaerobic conditions, confirming its long-term persistence.^19,20^ Therefore, it is crucial to develop advanced detection systems capable of simultaneously identifying multiple pesticide residues to protect public health and environmental safety.^21,22^

Current techniques for CBZ detection include UV–visible spectroscopy, Raman spectroscopy, liquid chromatography-tandem mass spectrometry (LC–MS/MS), high-performance liquid chromatography (HPLC), fluorescence spectroscopy, and spectrofluorimetry.^23,24^ While these methods are highly accurate, they often require expensive equipment, laborious sample preparation, and extended analysis time. Thus, the need arises for a detection method that is fast, precise, affordable, sensitive, and easy to operate.^25^ Electrochemical sensing technologies offer numerous advantages, including low cost, rapid response, high sensitivity, and ease of miniaturization. Compared to conventional methods, they are more suitable for real-time, on-site analysis and are increasingly favored in pesticide detection research.^2,26^ Electrochemical sensors have proven to be effective for detecting CBZ due to their simplicity, low detection limits, high reproducibility, and strong anti-interference properties.^27^

Transparent conductive oxides (TCOs), such as ZnO, In_2_O_3_:Sn, and In_2_O_3_:Mo, are commonly used in optoelectronic devices, including solar cells, flat-panel displays, and touchscreens. Delafossite-type materials, with the general formula AB_2_, have attracted increasing attention due to their tunable band gaps, p-type conductivity, and excellent optoelectronic characteristics.^28,29^ Compounds such as CuAlO_2_, CuGaO_2_, CuFeO_2_, CuRhO_2_, CuCoO_2_, and CuMnO_2_ have been studied for applications in photocatalysis, transparent electrodes, gas sensors, and solar cells.^30,31^ Among them, CuAlO_2_ stands out because of its low cost, excellent electrical conductivity, high transparency, thermal stability, and corrosion resistance.^32,33^

Since its discovery as a p-type transparent conducting oxide by Kawazoe et al. in 1997, CuAlO_2_ has been studied for use in transparent electronics, luminous materials, and electrochemical sensors due to its wide band gap (∼3.5 eV) and high room-temperature conductivity (∼1 S·cm^–1^).^34^ Several synthesis routes have been developed to obtain CuAlO_2_ nanoparticles, including ion exchange, hydrothermal methods, sol–gel processes, and high-temperature solid-state reactions.^35^ CuAlO_2_ possesses tunable optoelectronic properties, is environmentally benign, and is nontoxic, making it a suitable candidate for modifying electrochemical sensor electrodes.^36^

Structurally, CuAlO_2_ belongs to the R3̅m space group and features an anisotropic, quasi-two-dimensional framework composed of alternating Cu ion layers and AlO_2_ octahedra. Each Cu atom forms a linear coordination with two oxygen atoms along the *c*-axis, creating O–Cu–O dumbbell structures that bridge Cu and AlO_2_ layers.^37,38^ This leads to a multilayered, quasi-two-dimensional superlattice architecture. Two main crystal arrangements are observed depending on the stacking of AlO_6_ octahedral layers, including a hexagonal 2H configuration with *P*6_3_/*mmc* symmetry.^39–41^ The resulting material exhibited a well-ordered hierarchical helical structure, a large specific surface area, efficient electron transfer capability, and superior electronic conductivity.^42^

The fabricated GCE@CuAlO_2_ electrode was employed for the simultaneous electrochemical detection of CBZ. Compared to existing CBZ sensors, the GCE@CuAlO_2_ sensor demonstrated improved sensitivity, reproducibility, and long-term stability. The CBZ oxidation peak current was highly responsive to its concentration. The electrochemical performance of the sensor was thoroughly evaluated using differential pulse voltammetry (DPV), cyclic voltammetry (CV), and amperometric methods (it). The modified GCE@CuAlO_2_ electrode exhibited a wide linear concentration range, ultralow detection limit, high anti-interference ability, excellent repeatability, long-term stability, and accurate performance in real sample analysis.

## 2 Experimental Section

### 2.1 Materials

Copper nitrate trihydrate [Cu­(NO_3_)_2_·3H_2_O, ≥ 99.0%], aluminum nitrate nonahydrate [Al­(NO_3_)_3_·9H_2_O, ≥ 99.0%], carbendazim (CBZ) (C_9_H_9_N_3_O_2_), ethanol (C_2_H_5_OH), methanol (CH_3_OH), glycine (C_2_H_5_NO_2_), sodium phosphate monobasic (NaH_2_PO_4_·H_2_O), sodium phosphate dibasic monohydrate (Na_2_HPO_4_), sulfuric acid (H_2_SO_4_), and hydrochloric acid (HCl) were all procured from Sigma-Aldrich, Taiwan. Sodium phosphate monobasic and dibasic were used to prepare the phosphate buffer solution (PBS; 0.05 M), which was prepared using deionized water. The pH of the buffer solution was adjusted using 0.1 M H_2_SO_4_ or NaOH, and the pH values were monitored and maintained using a calibrated precision pH meter (PHS-3C, Leici Devices Factory of Shanghai, China). All other chemicals used were of analytical reagent (AR) grade and used without any further purification. Anhydrous CH_3_OH was used to prepare the stock solution of CBZ at a concentration of 1 μM. All experimental solutions were prepared using double-distilled water. Water purification was achieved using a Millipore system, ensuring a resistivity of 18.2 MΩ·cm. The ultrasonic treatment of materials was performed using an ultrasonic cleaner (KQ-100E, 100W, Kunshan, China), while the laboratory samples were prepared using an EMAG ultrasonic cleaner from the HC Pro series. The pH meter was calibrated daily using standard buffer solutions to ensure accuracy.

### 2.2 Characterization Methods

The crystalline structure of the synthesized material was examined using X-ray diffraction (XRD) on a PANalytical X’PERT PRO diffractometer (Netherlands), utilizing Cu Kα radiation (λ = 1.5417 Å). The vibrational functional groups were identified using Fourier Transform Infrared (FTIR) spectroscopy, conducted on an FTIR-6600 spectrometer in the range of 4000–400 cm^–1^, to confirm the molecular structure of the material during synthesis. Raman spectra were obtained using a LabRAM HR Raman spectrometer equipped with a 532 nm laser source. The oxidation states of elements (Cu, Al, O, and C) present in the CuAlO_2_ structure were analyzed by X-ray Photoelectron Spectroscopy (XPS) using Thermo Scientific MultiLab 2000 equipment. Morphological characteristics and surface textures of the synthesized samples were investigated using High-Resolution Transmission Electron Microscopy (HR-TEM; JEM 2100F), Energy-Dispersive X-ray Spectroscopy (EDX), and Scanning Electron Microscopy (SEM; JEOL JSM-6500F). Electrochemical Impedance Spectroscopy (EIS) was carried out using a ZAHNER impedance analyzer (Kroanch, Germany), operating in a frequency range of 100 mHz to 100 kHz. The EIS studies were performed in a solution containing 0.1 M KCl and 5.0 mM [Fe­(CN)_6_]^3–/4,^ with a fixed potential of 150 mV and a frequency range of 10–100 Hz. The electrochemical properties of the synthesized CuAlO_2_ nanomaterials and modified electrodes were further investigated using Cyclic Voltammetry (CV), Differential Pulse Voltammetry (DPV), and Electrochemical Impedance Spectroscopy (EIS) employing CHI1205b and CHI900 electrochemical workstations. A standard three-electrode setup was used for all electrochemical measurements. The working electrode was a glassy carbon electrode (GCE, φ = 3 mm, geometric area = 0.0729 cm^2^), the counter electrode was a platinum (Pt) wire, and the reference electrode was an Ag/AgCl electrode saturated with KCl (SCE). All electrochemical experiments were performed in a 0.05 M PBS buffer solution saturated with high-purity nitrogen gas at room temperature. Prior to each measurement, nitrogen was purged into the electrolyte solution (100 mM, pH 4.0) to eliminate dissolved oxygen, and this process was maintained continuously throughout all electrochemical runs. The optimized geometry and electronic properties of the CBZ molecule have been obtained using Gaussian 09 software and the Quantum ESPRESSO package. The limit of detection (LOD) for CBZ detection was determined using the formula in eq 1

LOD=3σ/m 1

where σ is the standard deviation of the blank signal and m is the slope of the calibration curve.

### 2.3 Preparation of CuAlO_2_ Nanopowder

The delafossite-type CuAlO_2_ nanopowder was synthesized by a glycine-nitrate process (GNP), a solution combustion method. The starting precursors were copper nitrate trihydrate [Cu­(NO_3_)_2_·3H_2_O], aluminum nitrate nonahydrate [Al­(NO_3_)_3_·9H_2_O], and glycine [C_2_H_5_NO_2_] as a fuel. The copper nitrate to glycine molar ratios were maintained at 1:1.5 and 1:1.7, corresponding to G/N ratios of 1.5 and 1.7, respectively. The molar ratio of copper nitrate to aluminum nitrate was fixed at 1:1. Equal weights of copper and aluminum nitrates (2.5 g each) were dissolved in distilled water to obtain a clear sky-blue solution. Subsequently, 1.125 g of glycine was added to the solution, and the mixture was continuously stirred at 80 °C for 45 min to yield a homogeneous and transparent solution. This precursor solution was then aged at 100 °C for 24 h in a 500 mL beaker to facilitate the gradual evaporation of water and the development of a viscous gel-like material. The gel was then heated on a hot plate to approximately 300 °C, which initiated spontaneous combustion, resulting in the formation of CuAlO_2_ nanopowder. During combustion, a gray foamy mass was formed, accompanied by the evolution of nitrogen dioxide (NO_2_) gas. To prevent the escape of nanopowder during the combustion process, a fine-mesh screen was placed over the beaker. After combustion, the obtained grayish nanopowder was dried for 48 h at room temperature. The synthesized CuAlO_2_ nanopowder was then collected, characterized, and stored for further use in electrochemical applications (eq 2).

$$\mathrm{Cu}({\mathrm{NO}}_{3}{)}_{2}+\mathrm{Al}({\mathrm{NO}}_{3}{)}_{3}+C_{2}H_{5}{\mathrm{NO}}_{2}\to {\mathrm{CuAlO}}_{2}+{\mathrm{CO}}_{2}+H_{2}O+N_{2}+{\mathrm{NO}}_{x}$$ 2

### 2.4 Pretreatment of Glassy Carbon Electrodes (GCE) for Electrochemical Sensors

Glassy carbon electrodes are frequently used in electrochemical sensing systems due to their superior electrical conductivity, chemical inertness, broad electrochemical window, and minimal background current. Nonetheless, the surface condition of these electrodes significantly influences their electrochemical performance. Therefore, an appropriate surface treatment is necessary to enhance reproducibility, sensitivity, and overall analytical reliability. The treatment process typically begins with mechanical polishing, where the GC electrode surface is abraded using a fine alumina slurry (approximately 0.05 μm) on a soft polishing pad to remove contaminants and surface irregularities. Following this, the electrode is thoroughly rinsed with distilled water and then ultrasonicated in both deionized water and ethanol to ensure the complete removal of any remaining particles or polishing residues. Subsequently, the electrode undergoes electrochemical activation by CV in an acidic solution, such as 0.5 M sulfuric acid. The potential is swept between −1.0 V and +1.5 V vs Ag/AgCl for multiple cycles. This step introduces oxygen-containing functional groups onto the surface, improving electron transfer and enhancing sensor response. Pretreatment is a vital step in preparing GCE for efficient use in electrochemical sensors. Once the surface is properly prepared, the electrode is ready for further modification and integration into sensor systems.

### 2.5 Preparation of the Modified GCE with CuAlO_2_ (GCE@CuAlO_2_)

The glassy carbon electrode (GCE) was modified with the synthesized CuAlO_2_ nanopowder for use in electrochemical sensing studies. Prior to modification, the GCE was polished sequentially using alumina slurry (1.0 μm, 0.3 μm, and 0.05 μm), washed thoroughly with Millipore water and ethanol, and ultrasonicated to remove any remaining debris. A total of 20 mg of CuAlO_2_ nanopowder was dispersed in 20 mL of dimethylformamide (DMF), followed by sonication for 20 min at room temperature to ensure uniform dispersion. A volume of 8 μL of the resulting suspension was then drop-cast onto the clean surface of the GCE. The coated electrode was left to dry under ambient room conditions, resulting in the formation of a uniform CuAlO_2_ layer due to electrostatic stacking between the CuAlO_2_ and the GCE surface. This prepared GCE@CuAlO_2_ electrode was subsequently employed for the electrochemical detection of CBZ. The entire fabrication process of the GCE@CuAlO_2_ electrode is depicted schematically in Scheme 1, illustrating the sequential steps involved in sensor electrode preparation.

**Scheme 1:**
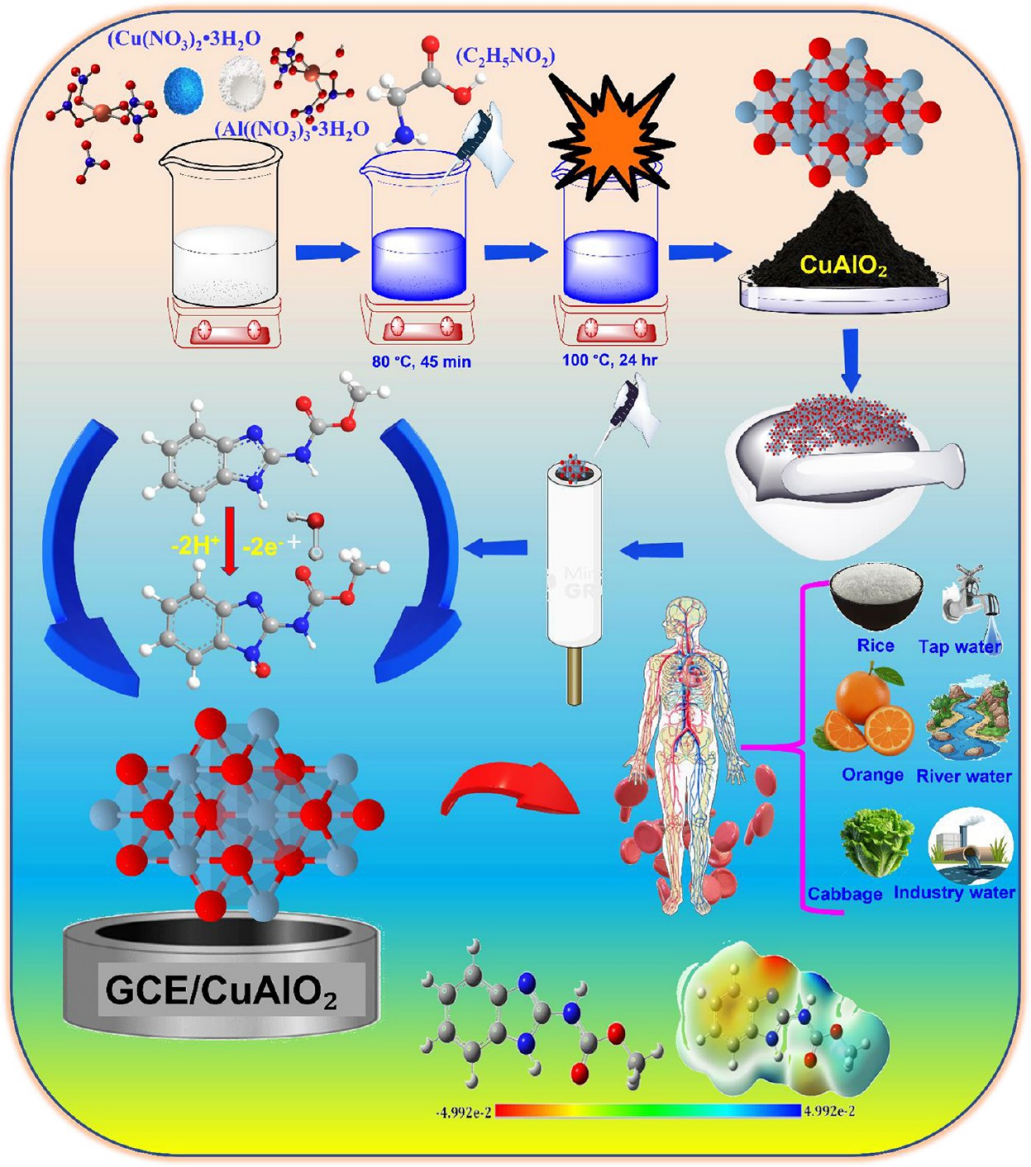
Schematic Illustration of GCE@CuAlO_2_ Electrode Preparation and Electrochemical Strategy for CBZ Sensor

## 3 Results and Discussion

### 3.1 XRD Characterization Studies

The crystal structures of CuAlO_2_ nanopowder catalysts obtained with glycine-to-nitrate (G/N) ratios of 1.0, 1.3, 1.5, and 2.0 were examined by powder X-ray diffraction (XRD) using a diffractometer equipped with crystal-database software. Although previous studies have shown that the glycine nitrate process (GNP) yields highly crystalline CuAlO_2_, the present work confirms that Cu and Al ions naturally favor the formation of delafossite-type CuAlO_2_. Figure 1 displays the XRD patterns for the four G/N ratios. All samples show five dominant reflections characteristic of the delafossite phase at 2θ ≈ 31.3°, 36.4°, 40.8°, 55.8°, and 62.4°, which correspond to the (006), (012), (104), (018), and (110) lattice planes, respectively. These peak positions match well with the standard database card PDF #75-2356, confirming that delafossite CuAlO_2_ is the principal phase and that no impurity phases are detectable. The peak intensities remain essentially unchanged as the G/N ratio increases, indicating that the glycine content does not alter the crystalline phase. All reflections can be indexed to the rhombohedral structure with space group $R\overset{®}{3}m$ . Structurally, the Cu atoms are linearly coordinated by two oxygen atoms, forming O–Cu–O dumbbell units that alternate with AlO_6_ edge-sharing octahedra. Within the delafossite lattice, Cu^+^ occupies the (0, 0, 0) site, Al^3+^ resides at (0, 0, 1/2), and oxygen is positioned at (0, 0, u). The prominent, sharp reflections indicate a high degree of crystallinity, and no peaks corresponding to potential contaminants, such as Al_2_O_3_ or CuO, are observed. The average crystallite size, *D*, was estimated with the Debye–Scherrer eq 3:

D=0.9λ/cosθ 3

where λ is the X-ray wavelength, θ is the Bragg angle, and β is the full width at half-maximum (fwhm) of the diffraction peak. The calculated crystallite sizes for CuAlO_2_ prepared at G/N = 1.0, 1.3, 1.5, and 2.0 are 15.02, 17.28, 14.23, and 13.61 nm, respectively.

**Figure 1:**
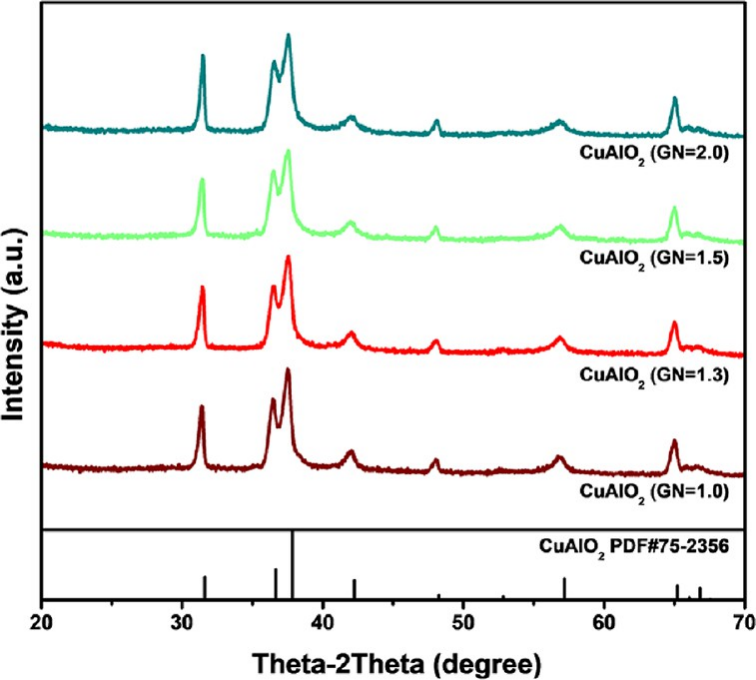
XRD studies of CuAlO_2_ (GN = 1.0), CuAlO_2_ (GN = 1.3), CuAlO_2_ (GN = 1.5), CuAlO_2_ (GN = 2.0).

### 3.2 Field-Emission Scanning Electron Microscopy (FESEM)

The surface morphologies of the CuAlO_2_ samples synthesized at G/N ratios of 1.0, 1.3, 1.5, and 2.0 were further inspected by FESEM (Figure 2A–P). All four specimens exhibit a sponge-like, highly porous architecture resembling amorphous cotton candy, an expected consequence of the rapid gas evolution during the self-combustion step of the GNP route. As the G/N ratio increases, the number of pores grows, while the overall crystallinity first improves and then diminishes. The simultaneous release of large volumes of gaseous products and the substantial reaction heat jointly enlarge the pore volume in the as-combusted powders. In addition, the CuAlO_2_ surfaces display well-defined, board-shaped crystalline domains that are consistent with the high-intensity reflections observed in the XRD patterns, further confirming the high phase purity and crystallinity of the delafossite structure.

**Figure 2:**
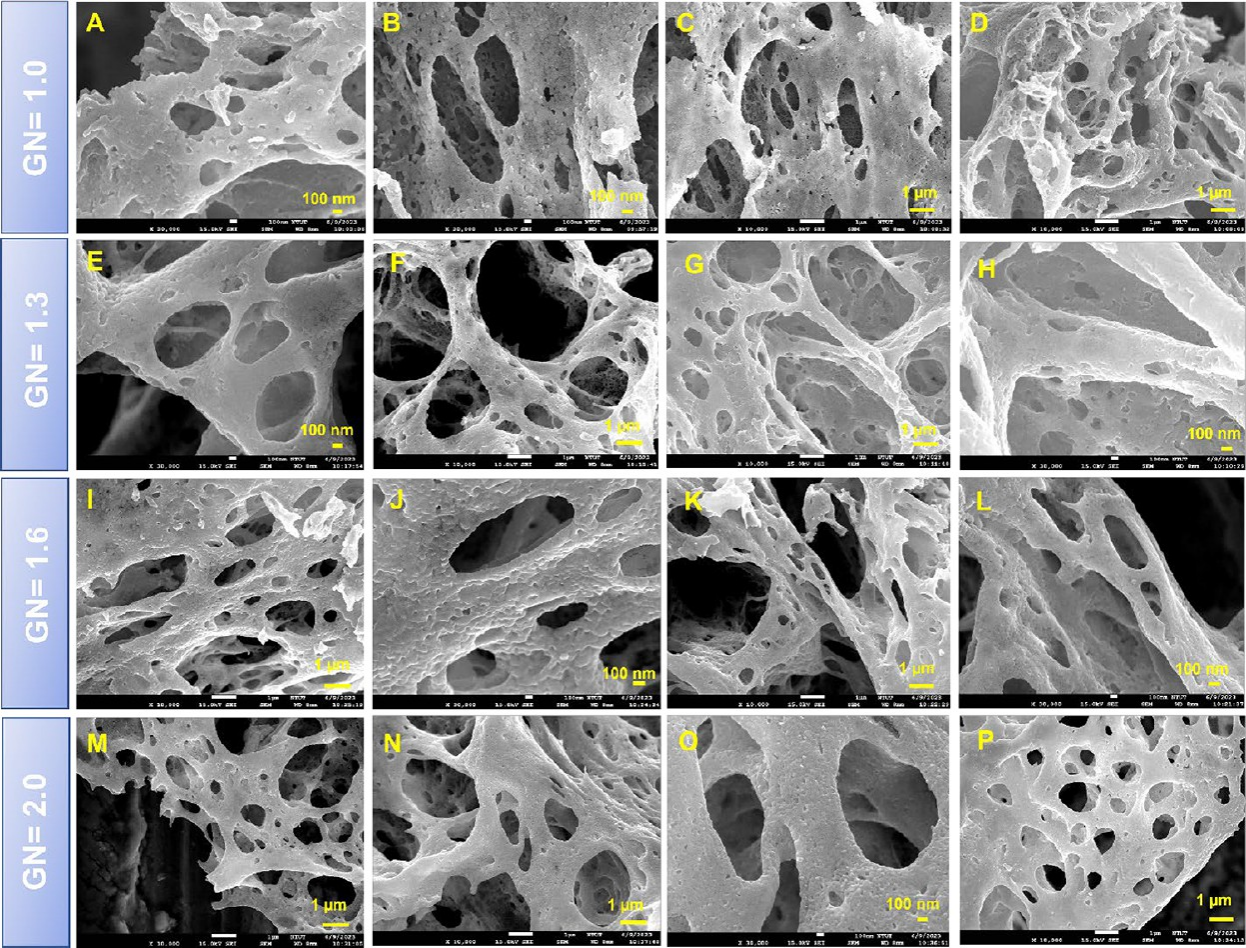
SEM images of (A–D) CuAlO_2_ (GN = 1.0), (E–H) CuAlO_2_ (GN = 1.3), (I–L) CuAlO_2_ (GN = 1.5), (M–P) CuAlO_2_ (GN = 2.0).

## 4 Morphology, Elemental Composition, and Spectroscopic Analyses

### 4.1 High-Resolution Transmission Electron Microscopy (HRTEM)

Figure 3A–I displays representative HRTEM micrographs of CuAlO_2_ synthesized at G/N = 2.0. The material forms rippled, ultrathin sheets with abundant folds and voids, imparting high transparency to the electron beam. Individual sheets have smooth surfaces and lateral dimensions of approximately 100–500 nm. The pronounced porosity and “curtain-like” texture arise from complete sintering and the vigorous evolution of oxygen and other gases during the self-combustion step.

**Figure 3:**
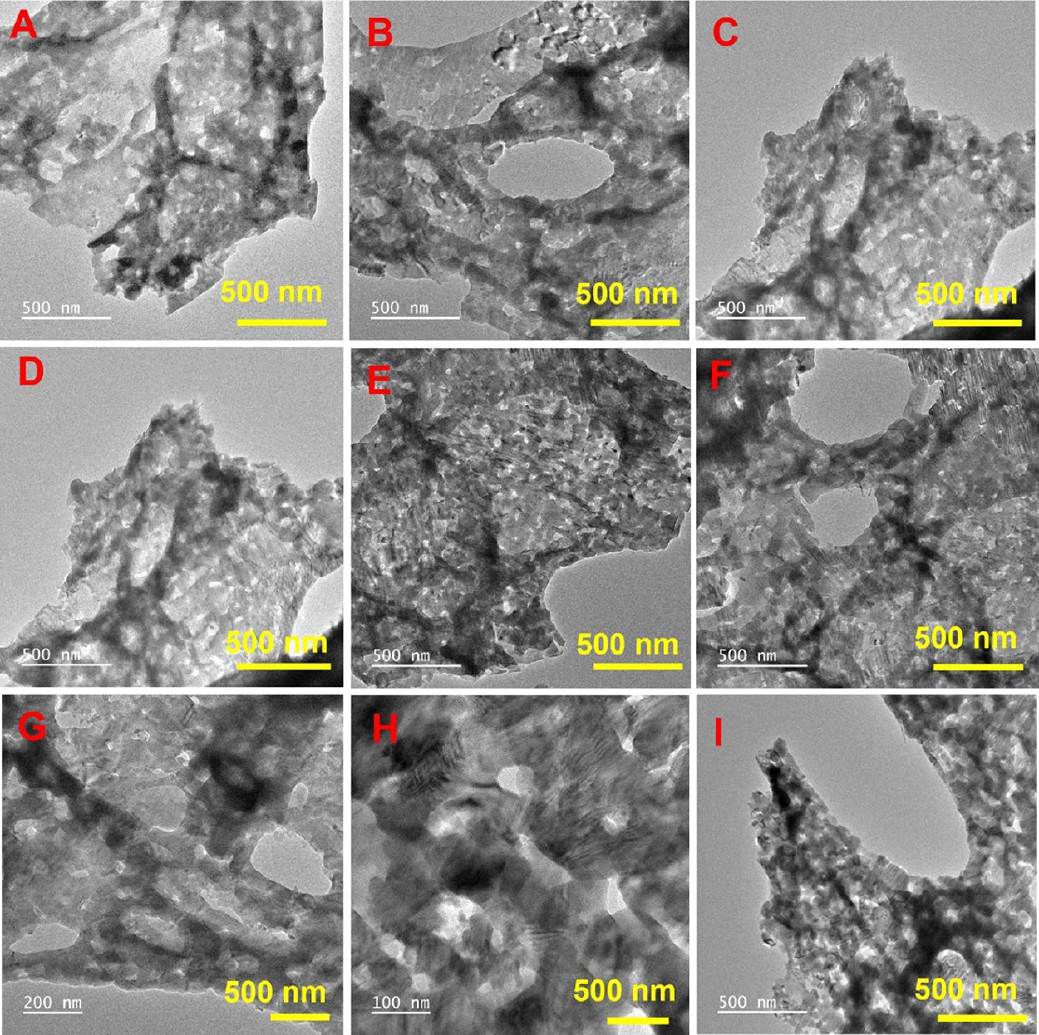
(A–I) HRTEM images of CuAlO_2_ (GN = 2.0) nanopowder catalysts.

### 4.2 Energy-Dispersive X-ray Spectroscopy (EDS) and Elemental Mapping

Figure 4 summarizes STEM-EDS results for the same G/N = 2.0 sample. Figure 4A,B presents STEM images of the selected region for analysis, revealing the same highly porous, curtain-like framework and amorphous regions, especially near edges or interfaces, as observed by HRTEM. Besides, no significant structural defects are observed, indicating high crystallinity. The image also exhibits uniform contrast, indicating a homogeneous composition without phase segregation. Figure 4C presents the combined elemental map analysis. In contrast, Figures 4D–F show individual maps for Cu, Al, and O. All three elements are homogeneously distributed, confirming uniform incorporation of Cu^+^, Al^3+^, and O^2–^ within the delafossite lattice and the absence of extraneous phases. Figure 4G shows the corresponding EDS spectrum, which exhibits peaks only for Cu, Al, and O. Quantitative analysis yields atomic percentages of 28.81 ± 0.04% (Cu), 22.13 ± 0.07% (Al), and 49.07 ± 0.15% (O) and weight percentages of 56.98 ± 0.09% (Cu), 18.58 ± 0.06% (Al), and 24.44 ± 0.08% (O); these values agree well with the theoretical stoichiometry of CuAlO_2_ (Cu: Al: O ≈ 1:1:2). Moreover, HRTEM and STEM images are concordant with previously reported work.

**Figure 4:**
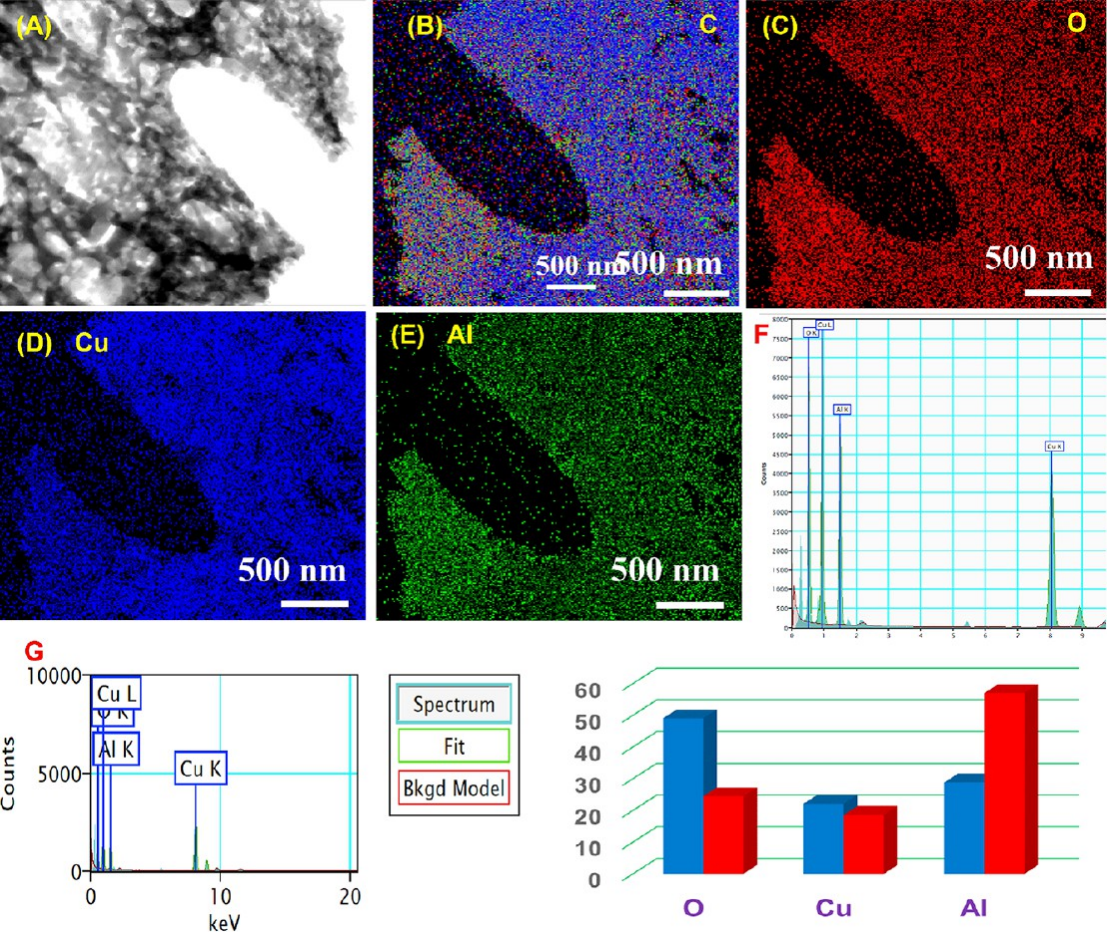
(A) STEM images of CuAlO_2_ (GN = 2.0) nanopowder catalysts, (B) elemental mapping analysis of CuAlO_2_ (GN = 1.3), elemental mapping of (C) O, (D) Cu and (E) Al and (F) EDX spectra with (G) elemental weight ratio of CuAlO_2_ (GN = 2.0) nanopowder catalysts.

### 4.3 X-ray Photoelectron Spectroscopy (XPS)

To probe surface composition and oxidation states, wide-scan XPS spectra were collected (Figure 5a). Signals at binding energies of ∼935 eV (Cu 2p), 75 eV (Al 2p), and 530 eV (O 1s) confirm the presence of Cu, Al, and O, respectively. High-resolution Cu 2p spectra (Figure 5a inset) reveal Cu 2p_3_/_2_ components at 932.8 and 934.2 eV, assignable to Cu^+^ and Cu^2+^, and Cu 2p_1/2_ components at 952.3 eV (Cu^+^) and 955.0 eV (Cu^2+^). The coexistence of Cu^+^ and Cu^2+^ is characteristic of delafossite oxides prepared by combustion routes. The Al 2p peak (Figure 5b) appears at 76 eV, indicative of Al^3+^ in an Al_2_O_3_-like environment; slight shifts toward lower binding energy at higher dopant concentrations reflect subtle changes in the AlO_6_ octahedra along the *ab* plane. The O 1s envelope (Figure 5c) deconvolutes into a lower-binding-energy component at 530.0 eV (Cu–O) and a higher-binding-energy component at 531.6 eV (Al–O). A faint shoulder at 532.2–532.8 eV grows with higher annealing temperature, consistent with hydroxyl or CuAl_2_O_4_-related oxygen species. Collectively, the XPS data verify the formation of phase-pure CuAlO_2_ with the expected oxidation states and without detectable impurities.

**Figure 5:**
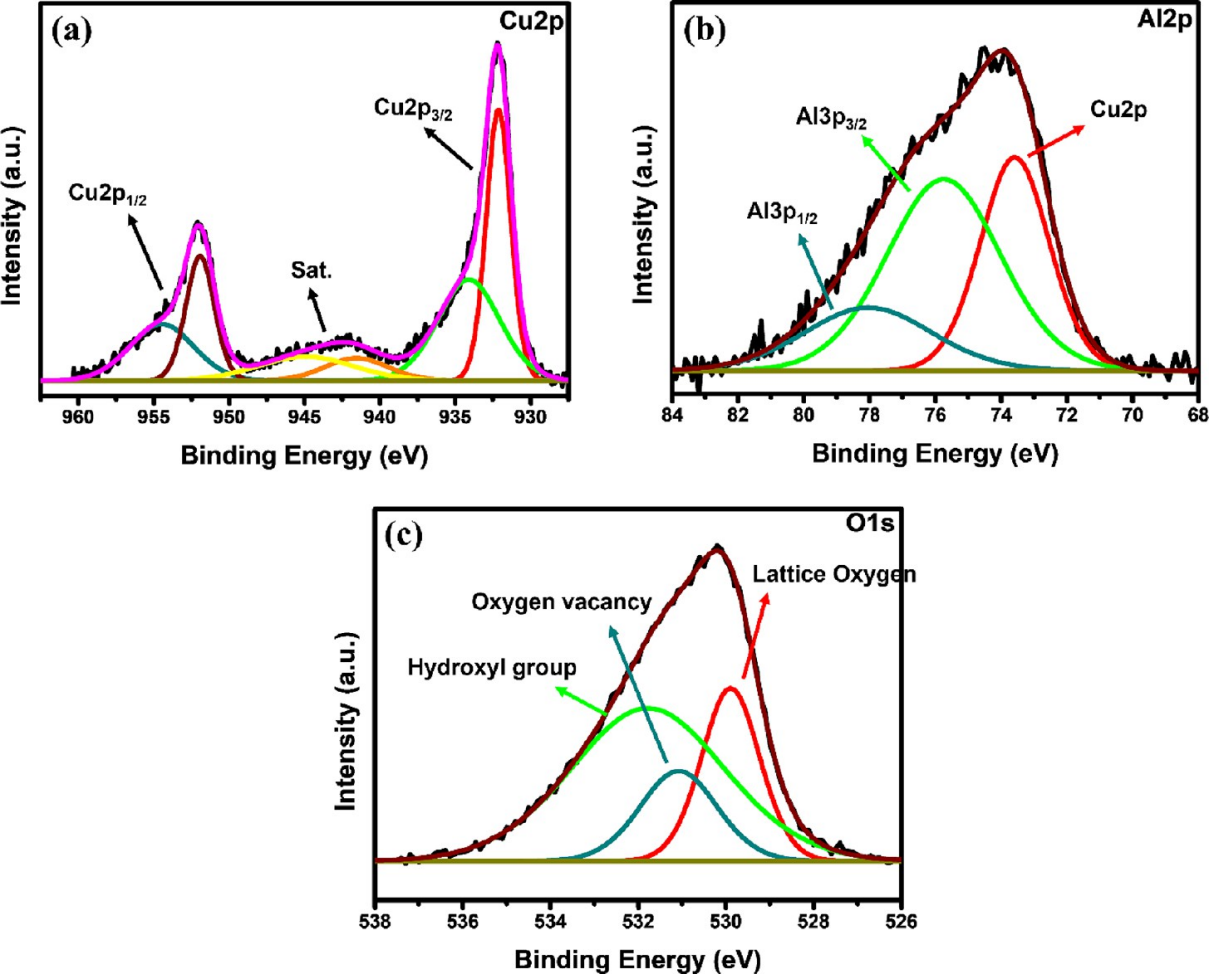
XPS spectra CuAlO_2_ nanoparticles (A) Cu, (B) Al, (C) O.

### 4.4 Raman Spectroscopy

Figure 6 compares the Raman spectra of CuAlO_2_ powders prepared at G/N = 1.0, 1.3, 1.5, and 2.0. All samples exhibit characteristic delafossite modes: low-frequency I_2_ and I_3_ vibrations at ∼250 and ∼300 cm^–1^ and higher-frequency A_1_g and E_9_ modes at ∼767 and ∼ 420 cm^–1^, respectively. With increasing G/N ratio, all Raman intensities have the same and sharpest A_1_g and E_9_ peaks. The variations in the G/N ratio likely influence factors such as crystal quality, defect density, particle size, or stoichiometry; however, the core delafossite structure of CuAlO_2_ persists, leading to the observation of the same fundamental Raman peaks. The consistently stronger A_1_g band, corresponding to Cu–O vibrations parallel to the *c* axis, suggests that all powders preferentially orient along the (001) direction, an observation in line with the XRD results. The evolution of Raman intensity thus corroborates the trends in crystallinity revealed by diffraction. It confirms that the combustion-synthesized CuAlO_2_ retains the intrinsic vibrational fingerprint of the delafossite lattice across all glycine-to-nitrate ratios.

**Figure 6:**
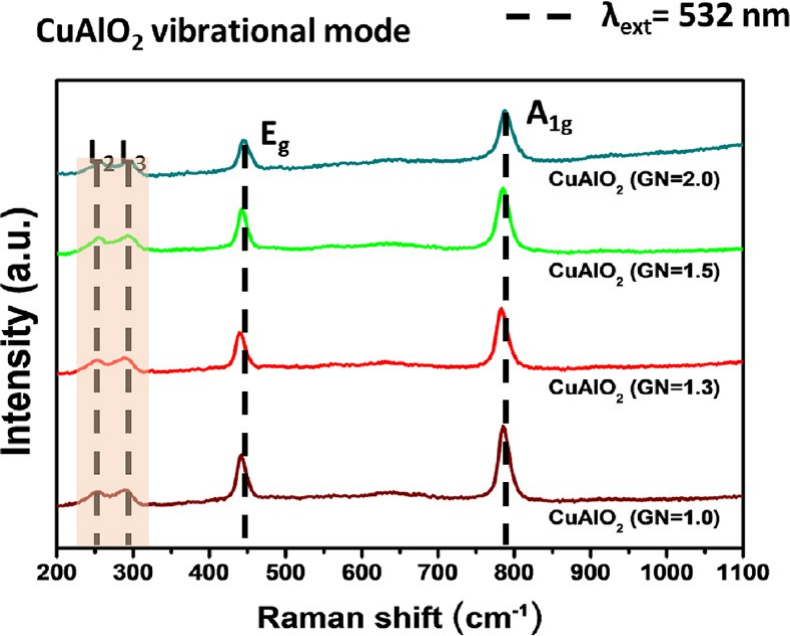
Raman spectra of CuAlO_2_ (GN = 1.0), CuAlO_2_ (GN = 1.3), CuAlO_2_ (GN = 1.5) and CuAlO_2_ (GN = 2.0).

### 4.5 Nitrogen Adsorption–Desorption and Surface Area Analysis

The nitrogen adsorption–desorption technique was employed to investigate the textural properties of CuAlO_2_ nanopowders synthesized with different glycine-to-nitrate (G/N) ratios: 1.0, 1.3, 1.5, and 2.0. The Brunauer–Emmett–Teller (BET) equation was applied to determine the specific surface areas of the samples, and the results are summarized in Table 1. The measured BET surface areas were 7.94 m^2^/g for CuAlO_2_ (GN = 1.0), 8.58 m^2^/g for CuAlO_2_ (GN = 1.3), 8.05 m^2^/g for CuAlO_2_ (GN = 1.5), and 5.92 m^2^/g for CuAlO_2_ (GN = 2.0). Among these, CuAlO_2_ (GN = 1.3) exhibited the highest surface area, suggesting that this composition yields a more favorable microstructure with a uniform distribution of pores and well-defined grain size.

**Table 1:** BET Surface Area of CuAlO_2_ (GN = 1.0, 1.3,1.5, and 2.0)

samples	specific surface area (m^2^/g)
CuAlO_2_ (GN = 1.0)	7.94
CuAlO_2_ (GN = 1.3)	8.58
CuAlO_2_ (GN = 1.5)	8.05
CuAlO_2_ (GN = 2.0)	5.92

The adsorption–desorption isotherms of all samples displayed clear hysteresis loops, which confirm the mesoporous nature of the CuAlO_2_ materials. The presence of such loops is indicative of capillary condensation occurring in pores with restricted geometries, commonly found in porous nanostructures. The trend in surface area reveals that moderate combustion temperatures promote enhanced porosity and surface development, as evidenced by the maximum BET area for GN = 1.3. However, further increases in combustion temperature, associated with GN = 2.0, resulted in significant sintering and subsequent pore collapse, leading to a sharp decline in surface area.

Additionally, the pore size distribution curves exhibited a gradual shift toward larger pore diameters, with a concurrent decrease in pore volume, particularly at higher G/N ratios. This behavior is consistent with particle consolidation and structural densification, which reduce the availability of open porosity. The microstructural transformation associated with excessive combustion results in the merging and collapse of smaller pores, thereby reducing the total surface area available for adsorption. The BET results suggest that fine control of the G/N ratio and combustion conditions is essential for tailoring the surface characteristics of CuAlO_2_ nanopowders for potential catalytic and adsorption applications.

### 4.6 Electrochemical Studies

#### 4.6.1 Electrochemical Impedance Spectroscopy Studies and Electrochemically Active Surface Area Finding

Electrochemical impedance spectroscopy (EIS) was employed to investigate the interfacial properties of the modified electrode, including polarization resistance (*R*_p_) and the electrical conductivity of the modified surface. Figure 7a displays the Nyquist plots of (a) bare GCE, (b) GCE/CuAlO_2_ (GN = 1.0), (c) GCE/CuAlO_2_ (GN = 1.3), (d) GCE/CuAlO_2_ (GN = 1.5), and (e) GCE/CuAlO_2_ (GN = 2.0) electrodes recorded in 0.1 M KCl containing 5 mM [Fe­(CN)_6_]^3–^/^4–^ over the frequency range of 100 mHz–100 kHz. Typically, a Nyquist plot consists of two distinct regions: a semicircular region, corresponding to the charge-transfer resistance (*R*_ct_), and a linear region, which reflects a diffusion-controlled process. The inset of Figure 7a illustrates the Randles equivalent circuit model, where *R*_ct_ denotes the charge-transfer resistance, *R*_s_ represents the electrolyte resistance, *Z*_w_ corresponds to the Warburg impedance, and *C*_dl_ indicates the double-layer capacitance. The calculated *R*_ct_ values for bare GCE, GCE/CuAlO_2_ (GN = 1.0), GCE/CuAlO_2_ (GN = 1.3), GCE/CuAlO_2_ (GN = 1.5), and GCE/CuAlO_2_ (GN = 2.0) were approximately 92, 86, 77, 73, and 70 Ω, respectively. Notably, the GCE/CuAlO_2_ (GN = 2.0) electrode exhibits a smaller semicircle diameter compared to all the other aforementioned electrodes, indicating enhanced electron transfer kinetics and reduced internal resistance. The EIS analysis revealed that the GCE/CuAlO_2_ (GN = 2.0) electrode exhibits superior conductivity and low resistivity, attributed to its efficient electron transfer characteristics and large surface area, which facilitate the sensing of CBZ.

**Figure 7:**
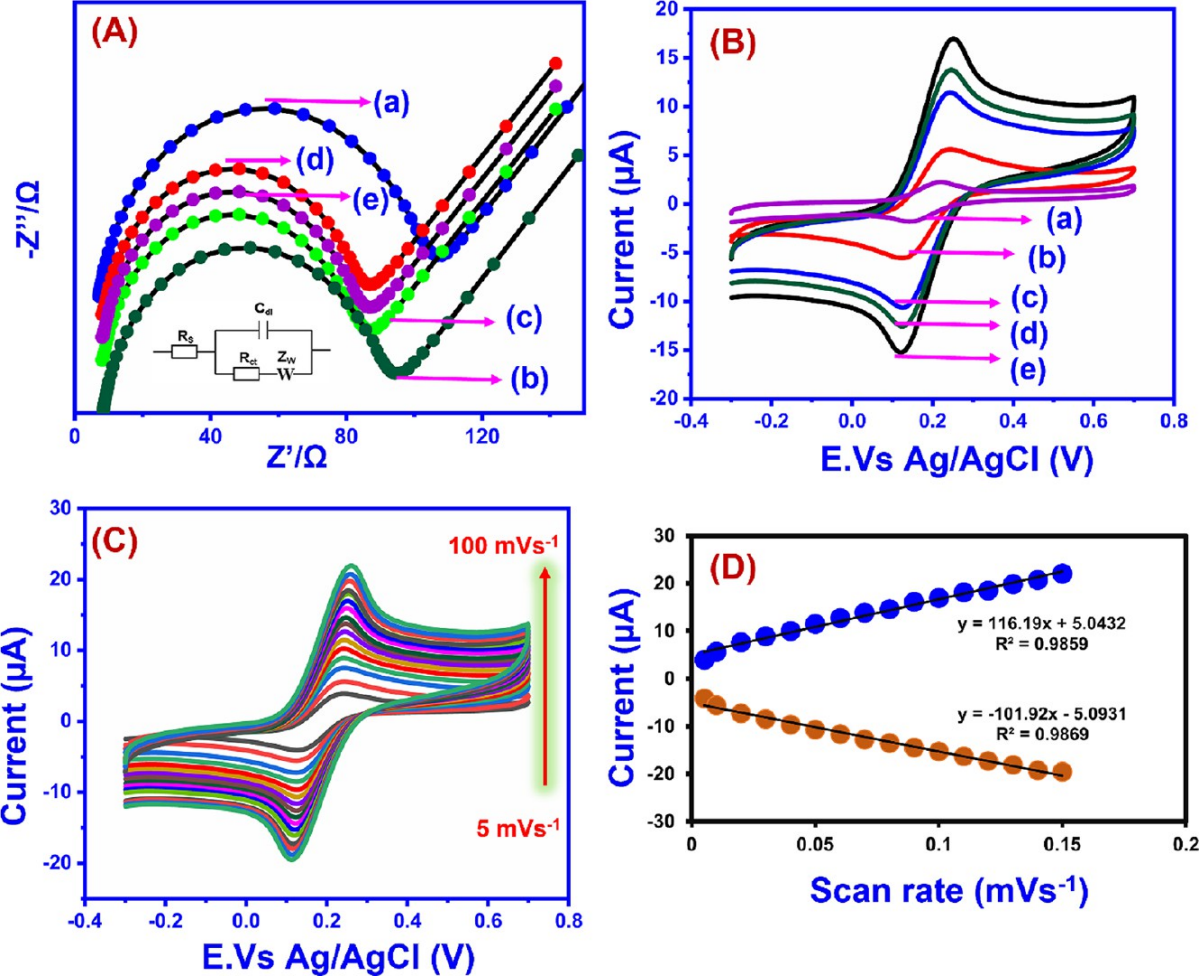
(A) EIS spectra of (a) GCE, (b) GCE/CuAlO_2_ (GN = 1.0), (c) GCE/CuAlO_2_ (GN = 1.3), (d) GCE/CuAlO_2_ (GN = 1.5), and (e) GCE/CuAlO_2_ (GN = 2.0) electrodes recorded in 0.1 M KCl containing 5 mM [Fe­(CN)_6_]^3–^/^4–^ over the frequency range 100 mHz-100 kHz. (B) CV responses of (a) GCE, (b) GCE/CuAlO_2_ (GN = 1.0), (c) GCE/CuAlO_2_ (GN = 1.3), (d) GCE/CuAlO_2_ (GN = 1.5), and (e) GCE/CuAlO_2_ (GN = 2.0) electrodes in 5 mM [Fe­(CN)_6_]^3–^/^4–^ with 0.1 M KCl. (C) CV curves of GCE/CuAlO_2_ (GN = 2.0) at different scan rates (5–100 mV s^–1^). (D) Linear relationship between scan rate and peak current (*I*_p_).

Figure 7b presents the CV profiles of (a) bare GCE, (b) GCE/CuAlO_2_ (GN = 1.0), (c) GCE/CuAlO_2_ (GN = 1.3), (d) GCE/CuAlO_2_ (GN = 1.5), and (e) GCE/CuAlO_2_ (GN = 2.0) electrodes recorded in 5 mM [Fe­(CN)_6_]^3–^/^4–^ containing 0.1 M KCl at a scan rate of 50 mV s^–1^. The electrochemically active surface area (ECSA) serves as a critical parameter, as it directly influences the kinetics of electron transfer and the overall efficiency of electrochemical processes at the electrode/electrolyte interface. Figure 7c displays the CV responses of the GCE/CuAlO_2_ (GN = 2.0) modified electrode recorded at scan rates ranging from 5 to 100 mV s^–1^, used to evaluate its electrochemically active surface area (ECSA). As shown in Figure 7d, the anodic and cathodic peak currents exhibit an excellent linear relationship with the scan rate, with correlation coefficients of *R* ^2^ = 0.986 for *I*_pa_ and *R* ^2^ = 0.985 for *I*_pc_. The ECSA was estimated using the Randles-Sevcik eq 4,

$$I_{p}=(2.69\times 105)n^{3/2}{AD}^{1/2}C\nu^{1/2}$$ 4

where “*I*_p_” represents the peak current, “*n”* is the number of electrons transferred, “*A”* is the active surface area, “*D”* is the diffusion coefficient, “*C”* is the concentration of ferricyanide, and “υ” is the scan rate. The electrochemically active surface areas of (a) bare GCE, (b) GCE/CuAlO_2_ (GN = 1.0), (c) GCE/CuAlO_2_ (GN = 1.3), (d) GCE/CuAlO_2_ (GN = 1.5), and (e) GCE/CuAlO_2_ (GN = 2.0) were determined to be 0.071, 0.25, 32, 0.33 and 0.35 cm^2^, respectively. Notably, the GCE/CuAlO_2_ (GN = 2.0) electrode exhibited both a lower charge-transfer resistance (*R*_ct_) and a larger active surface area, indicating superior electrical conductivity, enhanced electron-transfer kinetics, and excellent electrocatalytic activity.

#### 4.6.2 Differently Customized Electrodes for Electrochemical Reduction of CBZ

Figure 8A compares the cyclic-voltammetric responses of (a) bare GCE, (b) GCE@CuAlO_2_ (GN = 1.0), (c) GCE@CuAlO_2_ (GN = 1.3), (d) GCE@CuAlO_2_ (GN = 1.5), and (e) GCE@CuAlO_2_ (GN = 2.0) in 0.05 M PBS (pH 7.0) containing 100 μM CBZ, recorded at an identical scan rate of 50 mV s^–1^. In the absence of CBZ, the GCE@CuAlO_2_ (GN = 2.0) exhibits no discernible redox peaks, confirming that no background faradaic process occurs on this modified surface. For CBZ detection, the bare GCE exhibits only a weak anodic current of 20.89 μA at approximately 0.75 V, highlighting its inherently poor electrocatalytic activity. Introducing CuAlO_2_ markedly enhances the signal: GCE@CuAlO_2_ (GN = 1.0) yields an oxidation peak (*E*_p_ 0.77 V) with (*I*_p_) 34.64 μA. GCE@CuAlO_2_ (GN = 1.3) shifts the peak slightly to 0.79 V and increases (*I*_p_) to 38.41 μA. GCE@CuAlO_2_ (GN = 1.5) centers the peak at 0.78 V and further boosts (*I*_p_) to 52.08 μA. GCE@CuAlO_2_ (GN = 2.0) delivers the most pronounced response, with a sharp peak at 0.77 V and (*I*_p_) 61.85 μA, roughly triple the current obtained at bare GCE and significantly higher than any other CuAlO_2_ loading. Figure 8B shows the bar diagram of the peak current of GCE, GCE@CuAlO_2_ (GN = 1.0), GCE@CuAlO_2_ (GN = 1.3), GCE@CuAlO_2_ (GN = 1.5), and GCE@CuAlO_2_ (GN = 2.0) electrodes.

**Figure 8:**
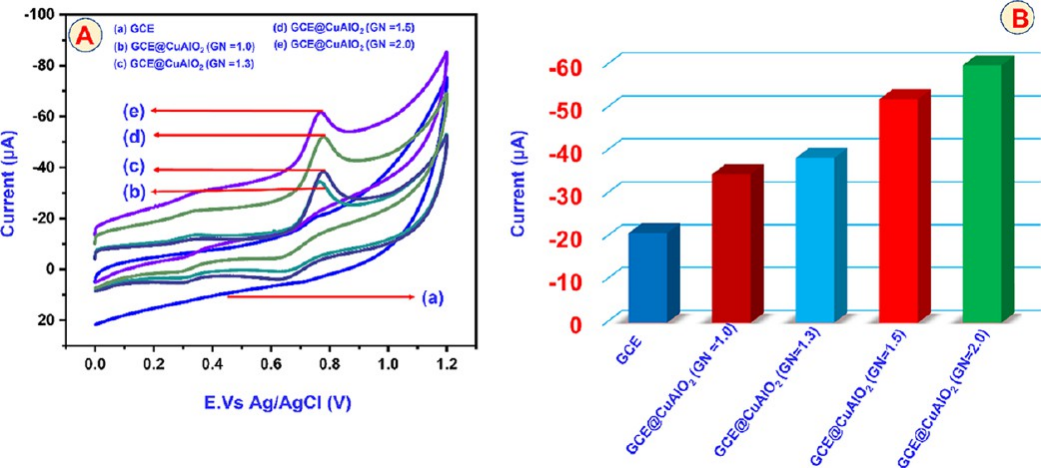
(A) Cyclic voltammetry performance of bare (a) GCE, (b) GCE@CuAlO_2_ (GN = 1.0), (c) GCE@CuAlO_2_ (GN = 1.3), (d) GCE@CuAlO_2_ (GN = 1.5), (e) GCE@CuAlO_2_ (GN = 2.0) electrodes in 150 μM CBZ containing PBS (pH 7) at a scan rate of 50 mV.s^–1^. (B) Bar diagram of the peak current of GCE, GCE@CuAlO_2_ (GN = 1.0), GCE@CuAlO_2_ (GN = 1.3), GCE@CuAlO_2_ (GN = 1.5), and GCE@CuAlO_2_ (GN = 2.0) electrodes.

The superior activity of the GN = 2.0 film can be attributed to two synergistic factors: (1) its higher electroactive surface area, which adsorbs more CBZ molecules, and (2) its excellent intrinsic conductivity, which accelerates electron transfer between the electrode and the analyte. Together, these features create abundant catalytic sites and foster rapid charge transport, thereby amplifying the CBZ oxidation signal. Electrochemically, CBZ is readily oxidized via two-electron/two-proton removal from the protonated nitrogen in the benzimidazole ring. The proposed electrocatalytic pathway at GCE@CuAlO_2_ (GN = 2.0) is summarized in Scheme 2, underscoring the role of the CuAlO_2_ film in facilitating electron–proton exchange. Overall, the data demonstrate that the GN = 2.0 composite provides outstanding catalytic enhancement for CBZ sensing, far surpassing the performance of both the bare electrode and CuAlO_2_ films prepared at lower G/N ratios.

**Scheme 2:**
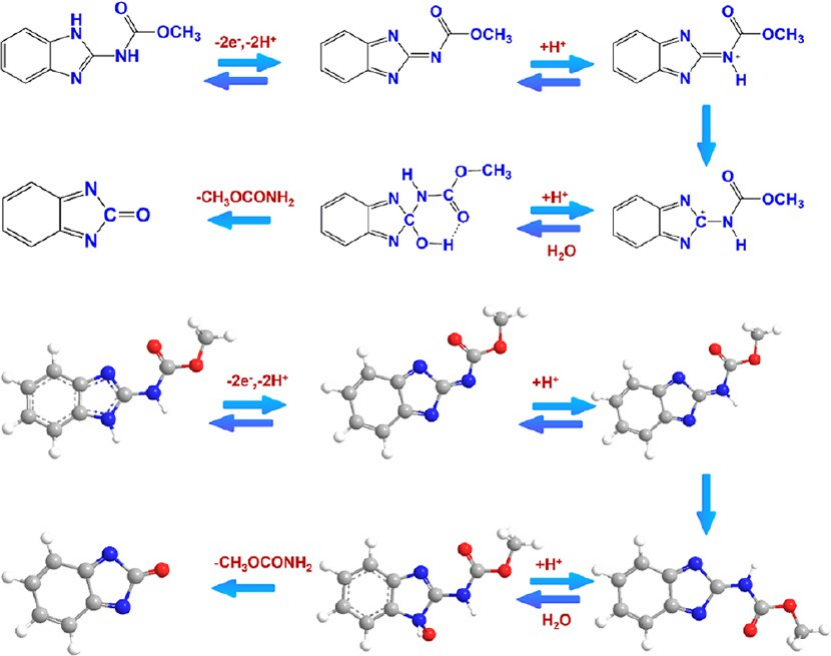
Electrocatalytic Reduction Mechanism of CBZ over at GCE@CuAlO_2_ (GN = 2.0) Electrodes

### 4.7 Impact of Different pH Investigations

The proton concentration of the electrolyte strongly affects the electro-oxidation behavior of CBZ; in alkaline media, CBZ is prone to hydrolytic degradation. CV studies were therefore performed with the GCE@CuAlO_2_ (GN = 2.0) electrode in 0.05 M PBS, adjusted from pH 3 to pH 11, each solution containing 20 μM CBZ, and scanned at 50 mV s^–1^. The resulting voltammograms are compiled in Figure 9A. As the pH is raised from 3 to 7, the anodic peak current (*I*_p_) increases steadily, reaching a maximum at pH 7.0; further alkalization (pH > 7) causes a gradual decline in current, consistent with diminished CBZ stability and slower proton-coupled electron transfer. Consequently, pH 7.0 was selected as the optimum working medium for all subsequent measurements.

**Figure 9:**
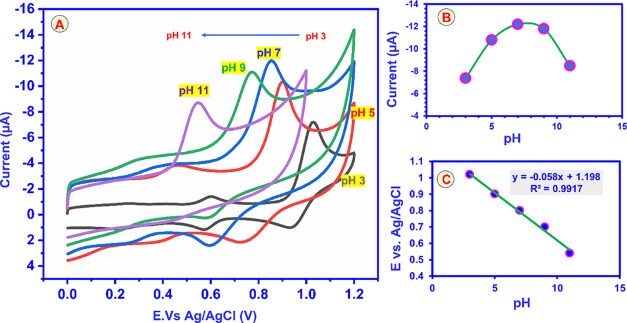
(A) Cyclic voltammograms of GCE@CuAlO_2_ (GN = 2.0) electrodes in PBS containing 20 μM of CBZ at various pH levels (3–11), (B) pH vs *I*_p_, (C) pH vs *E*_p_.

Figure 9B plots the corresponding oxidation peak potentials (*E*_p_) against pH. The potential shifts monotonically to more negative values with increasing pH, revealing direct participation of protons in the electrode process. A linear fit of *E*_p_ versus pH (Figure 9C) affords a slope close to the theoretical – 59 mV pH^–1^ predicted by the Nernst equation, indicating that the reaction proceeds with an equal number of electrons and protons. The proposed redox pathway (Scheme 2) involves a concerted two-electron/two-proton transfer in which the protonated nitrogen of the benzimidazole ring is oxidized to form a transient imidazole-2-yl radical and a methyl-carbamate fragment. Dimerization of the radical, followed by reduction, yields a benzimidazole alcohol derivative. Thus, the overall electrooxidation of CBZ at the GCE@CuAlO_2_ (GN = 2.0) surface can be described as a 2e^–^/2H^+^ process, fully consistent with the pH-dependent electrochemical data.

### 4.8 pH-Dependent Electrochemistry and Active-Area Evaluation

During electro-oxidation, CBZ undergoes coupled chemical (C) and electrochemical (E) steps, constituting an ECE mechanism. The pH dependence observed at the GCE@CuAlO_2_ (GN = 2.0) electrode confirms the participation of protons: as the solution pH increases from 3 to 11, the anodic peak potential (*E*_p_) shifts negatively. In contrast, the peak current (*I*_p_) decreases, reflecting slower proton-coupled electron transfer at higher alkalinity. The calibration plot of *E*_p_ versus pH (Figure 9C) is described by eq 5.

$$E_{p}=-0.058+1.198\mathrm{pH}\;(R^{2}=0.9917)$$ 5

which can be compared with the Nernst-type expression eq 6

$$E_{p}=\frac{0.0592M}{n}\mathrm{pH}+b$$ 6

The experimentally determined slope (≈ 59 mV pH^1^) yields an *m*/*n* ratio of 0.58, indicating that the oxidation of CBZ involves equal numbers of electrons and protons, consistent with the two-electron/two-proton pathway proposed in Scheme 2. Based on this, pH 7.0 was selected as the optimal medium for all subsequent analyses. Electrode active areas were estimated with the Randles–Ševčík (eq 7) using the reversible redox probe (K_3_[Fe­(CN)_6_]/K_4_[Fe­(CN)_6_] ([Fe­(CN)_6_]^3–^ + e^–^ ⇌ [Fe­(CN)_6_]^4–^).

$$I_{p}=2.69\times 105n^{3/2}{AD}^{1/2}Cv^{1/2}$$ 7

The GCE@CuAlO_2_ (GN = 2.0) electrode exhibited a significantly larger active surface area (0.186 cm^2^) compared to the GCE@CuAlO_2_ (GN = 1.0) (0.156 cm^2^), GCE@CuAlO_2_ (GN = 1.3) (0.142 cm^2^), and GCE@CuAlO_2_ (GN = 1.5) (0.121 cm^2^). Where *A* is the electroactive surface area. The calculated *A* values are 0.186 cm^2^ for GCE@CuAlO_2_ (GN = 2.0), markedly larger than those for GCE@CuAlO_2_ (GN = 1.0) at 0.156 cm^2^, GCE@CuAlO_2_ (GN = 1.3) at 0.142 cm^2^, and GCE@CuAlO_2_ (GN = 1.5) at 0.121 cm^2^. The enlarged active area of the GN = 2.0 film, together with its superior conductivity, accounts for its outstanding catalytic performance in CBZ detection.

### 4.9 Effect of Different Concentration Studies & Different Scan Rate Studies of GCE@CuAlO_2_ (GN = 2.0) Electrode

Figure 10A displays the cyclic voltammetry performance of the GCE@CuAlO_2_ (GN = 2.0) electrode by different concentrations of CBZ ranging from 10 μM to 320 μM in 0.05 M PBS (pH 7). The GCE@CuAlO_2_ (GN = 2.0) electrode is an effective electrode for the electrocatalytic oxidation of CBZ, as evidenced by the linear correlation between the CBZ oxidation peak current and CBZ concentration. Figure 10B illustrates the linear correlation between the CBZ peak current and varying concentrations of CBZ. The GCE@CuAlO_2_ (GN = 2.0) electrode was shown to exhibit a linear concentration range of up to 320 μM. The corresponding linear regression equation is given by *I*_pa_ = −0.1831 × [CBZ (μM)] – 43.571 with an *R* ^2^ value of 0.9921. The electrocatalytic oxidation of CBZ seems to follow first-order kinetics, as indicated by the slope value being nearly equal to 1.

**Figure 10:**
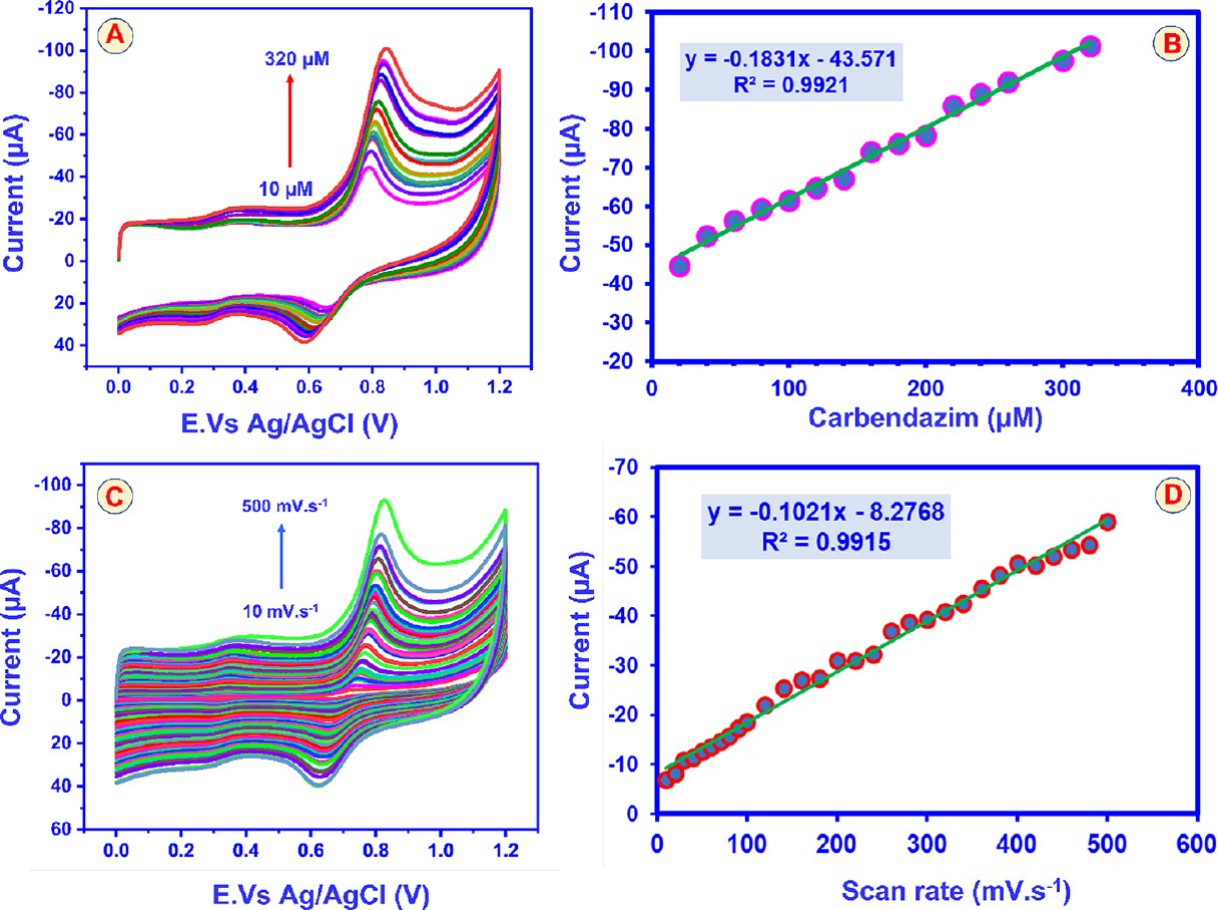
(A) Cyclic voltammetry responses to different concentrations of CBZ (10 μM to 320 μM) over GCE@CuAlO_2_ (GN = 2.0) in (1.0 pH) at 50 mVs^–1^. (B) Calibration plot of CBZ vs peak current. (C) CV response of the effect of different scan rates (10 to 500 mVs^–1^) 100 μM of CBZ detection on GCE@CuAlO_2_ (GN = 2.0), (D) corresponding linear plot of scan rate vs CBZ oxidation peak current.

Cyclic-voltammetric (CV) scans were first recorded at incremental sweep rates (not shown) to verify the kinetic regime of CBZ oxidation at the GCE@CuAlO_2_ (GN = 2.0) surface. The anodic peak current (*I*_p_) increased proportionally with the square root of the scan rate (*v* ^1/2^), confirming a diffusion-controlled, surface-confined process. Moreover, the linear shift of peak potential with log ν indicated quasi-reversible behavior, further validating the electrode’s rapid electron-transfer capability. Figure 10A depicts CV traces of GCE@CuAlO_2_ (GN = 2.0) in 0.05 M PBS (pH 7.0) upon successive additions of CBZ from 10 to 320 μM. The anodic peak grows systematically with concentration, underscoring the film’s excellent electrocatalytic activity. The calibration plot in Figure 10B shows a robust linear relationship between peak current (*I*_pa_) and CBZ concentration across the entire range examined. The regression eq 8 is

$$I_{\mathrm{pa}}(\mu A)=-0.1831\times [\mathrm{CBZ}(\mu M)]-43.571,R^{2}=0.9921$$ 8

Demonstrating outstanding linearity up to 320 μM. The near-unity slope (absolute value) indicates first-order kinetics with respect to CBZ, implying that the oxidation rate depends linearly on the substrate concentration under these conditions. Together with the scan-rate data, these results confirm that the GCE@CuAlO_2_ (GN = 2.0) electrode provides a sensitive, diffusion-controlled pathway for CBZ oxidation, thereby enabling accurate quantitative analysis over a broad concentration range.

To elucidate the kinetics and mechanism of CBZ oxidation on the GCE@CuAlO_2_ (GN = 2.0) surface, cyclic-voltammetric (CV) experiments were recorded at progressively higher scan rates (ν) from 10 to 500 mV s^–1^ in 0.05 M PBS (pH 7.0) containing 100 μM CBZ. The resulting voltammograms are compiled in Figure 10C. As ν increases, the anodic peak current (*I*_pa_) rises proportionally, and the peak potential (*E*_pa_) shifts to more positive values, a behavior consistent with the strong adsorption of CBZ onto the electrode and an irreversible electron-transfer process. Figure 10D presents the linear dependence of *I*_pa_ on ν over the entire sweep-rate window (10–240 mV s^–1^), described by eq 9

$$I_{\mathrm{pa}}(\mu A)=0.1021\nu \;({\mathrm{mVs}}^{-1})-8.2768,R=0.9915$$ 9

Confirming that the electrooxidation is adsorption-controlled. A complementary plot of *I*_pa_ versus ln ν (not shown) also yields a straight line, further indicating rapid electron transfer at the modified surface. The peak to potential shift obeys the Laviron formulation for an irreversible, adsorption-dominated process in eq 10

$$E_{\mathrm{pa}}=E^{0}+[RT/\alpha nF]\;\mathrm{In}[RTk^{0}/\alpha nF]+[RT/\alpha nF]\;\mathrm{In}(\nu )$$ 10

where *E* ^0^ is the formal potential, *R* = 8.314 J mol^–1^ K^–1^, *T* = 298 K, *F* = 96 480 C mol^–1^, α is the transfer coefficient, *n* is the number of electrons, and *k* ^0^ is the heterogeneous rate constant. From the slope of *E*_pa_ versus ln ν, the value of (*RT*/α*nF*) was extracted, yielding *n* ≈ 2 for α ≈ 0.5. Thus, the data corroborate a two-electron/two-proton (2e^–^/2H^+^) oxidation pathway, fully in accord with the mechanism outlined in Scheme 2. Overall, the scan-rate study confirms that CBZ oxidation at the GCE@CuAlO_2_ (GN = 2.0) electrode is adsorption-controlled, irreversible, and facilitated by fast electron transfer.

### 4.10 Amperometric Studies of CBZ Reduction over RRDE@CuAlO_2_ (GN = 2.0) Modified Electrode

Because amperometry combines a low background current with high selectivity at trace (nM–pM) levels, it was used to evaluate the CBZ-sensing capability of the CuAlO_2_ (GN = 2.0) nanocomposite. Figure 11A shows the current–time profile obtained at a CuAlO_2_-modified rotating ring–disc electrode (RRDE@CuAlO_2_, GN = 2.0) in nitrogen-purged 0.05 M PBS (pH 7.0). Successive injections of CBZ (0.01 – 800 μM) were made every 50 s while the disc rotated at 2500 rpm and the potential was held at – 0.25 V. Each addition produced an immediate, well-defined step in oxidation current that reached a steady state within ∼3 s, confirming the rapid electrocatalytic oxidation of CBZ on the composite surface. The calibration plot in Figure 11B relates the steady-state current (*I*_p_) to CBZ concentration and fits the linear regression eq 11

$$I_{p}(A)=0.4749\times [\mathrm{CBZ}(\mu M)]+7759\;\mathrm{and}\;R^{2}=0.9918$$ 11

**Figure 11:**
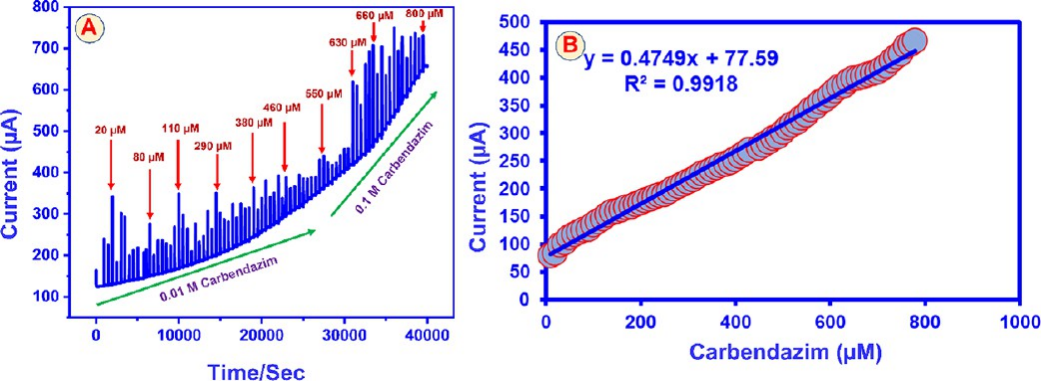
(A) Amperometric response of RRDE@CuAlO_2_ (GN = 2.0) electrode (*E*_app_ = −0.25 V) for oxidation of different additions of CBZ in PBS (pH = 7). (B) Linear plot of peak current versus CBZ concentration.

From the slope (*S* = 0.4749 A μM^–1^) and the standard deviation of the blank signal (σ_b_), the limit of detection (LOD) was derived as eq 12

LOD=3Sb/S 12

Sensor sensitivity, calculated as *S* divided by the geometric disc area (0.33 cm^2^), is 1.44 μA μM^–1^ cm^–2^. The device exhibits a broad linear dynamic range from 0.01 to 800 μM and 1 nM limit of detection a prompt, reproducible amperometric response for every CBZ aliquot. The exceptional behavior of RRDE@CuAlO_2_ (GN = 2.0) stems from several synergistic factors: (1) Homogeneous Electron-Rich Matrix: The nanocomposite’s conductive network fosters rapid charge transfer, lowering the CBZ oxidation overpotential and suppressing interference. (2) Large Electroactive Surface: The high surface area provides an abundance of catalytic sites, thereby enhancing mass transport and the adsorption of CBZ molecules. (3) Intrinsic Catalytic Activity: CuAlO_2_ (GN = 2.0) accelerates the two-electron/two-proton oxidation pathway, delivering high current efficiency. Overall, the RRDE@CuAlO_2_ (GN = 2.0) electrode significantly outperforms many previously reported CBZ sensors; comparative data are compiled in Table 2. These findings confirm that the CuAlO_2_ (GN = 2.0) nanocomposite provides a robust platform for ultrasensitive, rapid, and wide-range amperometric detection of carbendazim.

**Table 2:** Performance Comparison of Various Modified Electrodes with GCE@CuAlO_2_ (GN = 2.0) Electrode for CBZ Sensor

material	linear range	LOD	sensitivity (μA μM^‑1^ cm^2^)	references
^a^La–Nd_2_O_3_/CPE	0.08–50	0.027		^43^
^b^SiO_2_/MWCNT/GCE	0.2–4.0	0.056		^44^
^c^NGO/GCE	0.04–7.13	0.0084		^5^
^d^Ti_3_C_2_T_*x*_ Mxene/GCE	0.05–100.0	0.0103		^7^
^e^ZnFe_2_O_4_/SWCNTs/GCE	0.5–100.0	90		^45^
^f^Ru–Asp–Arg–GQD/GCE	0.01–45	0.004		^46^
^g^MWNT-PMRE	0.20–10.0	0.009		^47^
^h^Ce-doped ZnWO4/CPE	0.01–5.5	0.0033		^48^
^i^Nd_2_Mo_3_O_9_/MWCNTs/GCE	50–90	16.7	95.71	^49^
^j^GdW	0.02–40.00	5.0		^50^
^k^LaV/h-BN	0.001–89	0.5		^51^
^l^Mo_2_C@NiMn-LDH	0.001–232.14	0.2		^52^
^m^PrCoO_3_/RGO	0.001–84	0.002		^53^
^n^CPE/Fs@Ag	0.05–3.0	0.94		^54^
^o^Na montmorillonite clay/GCE		0.96		^55^
^p^NP-Cu/RGO/GCE	0.5–30	0.09		^56^
^q^BBD electrode	0.5–15	0.12		^57^
GCE@CuAlO_2_ (GN = 2.0)	0.01–800 μM	1 nM	1.44 μA μM^–1^ cm^–2^	this work

a La-dopedNd_2_O_3_.

b Mesoporoussilica/multiwalled carbon nanotubes.

c Electrochemicallyreduced nitrogen-doped graphene oxide-modified glassy carbon electrode.

d Ti_3_C_2_T_x_(MXene) and electrochemically reduced graphene oxide.

e ZnFe_2_O_4_/SWCNTs/GCE.

f Ruthenium-graphenequantum dot hybrid via the reduction of RuCl3 with aspartic acid andarginine-functionalized graphene quantum dots.

g Multiwalledcarbon nanotubes-polymeric methyl red film modified electrode.

h Ce-dopedZnWO_4_ modified carbon paste electrode.

i Neodymiummolybdate wrapped with multiwalled carbon nanotubes.

j Gadoliniumtungstate nanoflakes.

k Lanthanumvanadate/hexagonal boron nitrideLaV/h-BN.

l Molybdenumcarbide (Mo_2_C) MXene on three-dimensional Globe Amaranth flower-likeNiMn layered double-hydroxide (NiMn-LDH).

m Praseodymiumcobalt oxide.

n Silvernanoparticles on fumed silica

o Sodiummontmorillonite clay.

p Three-dimensionalnanoporous copper reduced graphene oxide glassy carbon electrodeNP-Cu/RGO/GCE.

q Boron-dopeddiamond electrode.

### 4.11 Selectivity, Operational Stability, and Cyclic Stability Studies of GCE@CuAlO_2_ (GN = 2.0) Electrode

A key requirement for practical electrochemical sensing is immunity to interference from coexisting species. The selectivity of the GCE@CuAlO_2_ (GN = 2.0) electrode was therefore challenged with a broad panel of structurally related antibiotics, pesticides, aromatic compounds, and common inorganic ions (Figure 12). Figure 13A depicts amperometric responses recorded after sequential additions of (a) CBZ, followed by large excesses of (b) furaltadone, (c) nitrofurazone, (d) 4-nitroaniline, (e) 4-nitrophenol, (f) chlorpyrifos, (g) metribuzin, (h) 4-aminophenol, (i) methyl-parathion, (j) Hg^2+^, (k) Pb^2+^, (l) Cu^2+^, (m) Cr^2+^, (n) I^–^, and (o) NO_3_^–^. Only CBZ produced a pronounced current step, whereas all other additives elicited negligible signals, even at concentrations far exceeding that of the target analyte. The peak-current variation remained below 2.56%, demonstrating that the GCE@CuAlO_2_ film effectively repels interferents by electrostatic forces (schematically illustrated in Figure 11) and maintains a highly selective response toward CBZ in complex matrices typical of water, food, and environmental samples.

**Figure 12:**
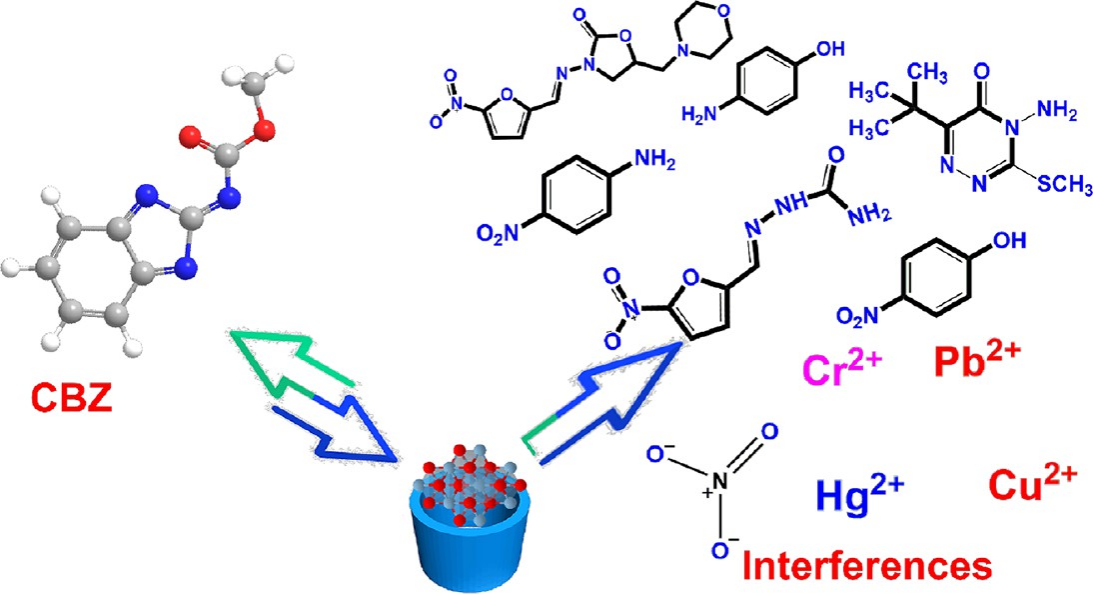
Schematic image of the effect of interfering substances on the electrochemical detection of CBZ over GCE@CuAlO_2_ (GN = 2.0) electrode.

**Figure 13:**
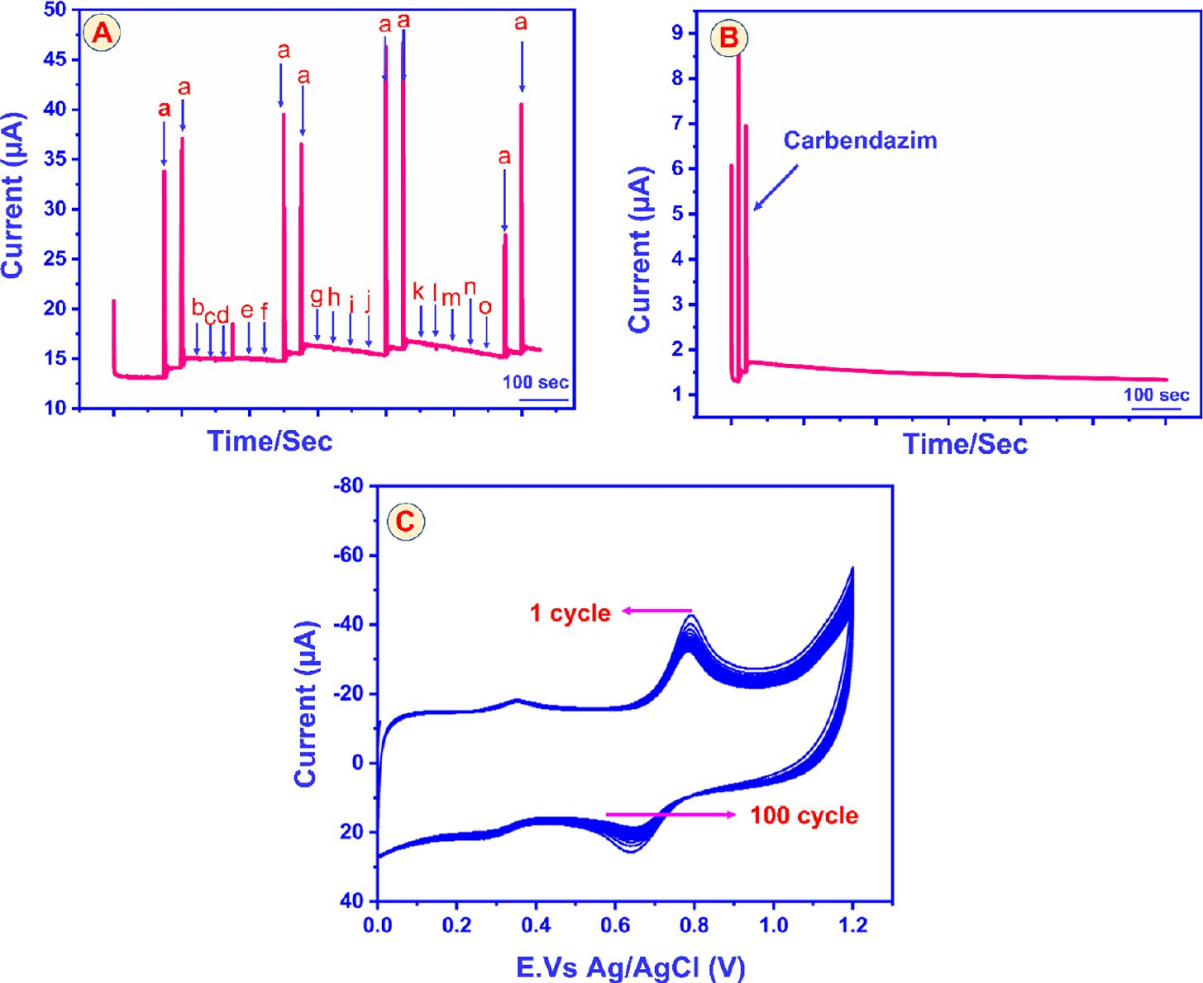
(A) Interference studies of GCE@CuAlO_2_ (GN = 2.0) electrode toward detection of CBZ (50 μM), ((a) CBZ, (b) furaltadone, (c) nitrofurazone, (d) 4-nitroaniline, 4-nitrophenol, (f) chlorpyrifos, (g) metribuzin, (h) 4-aminophenol, (i) methyl parathion, (j) mercury, (k) lead, (l) Cu^2+^, (m) Cr^2+^, (n) I^–^, and (o) NO_3_^–^). (B) Amperometric operational stability responses of GCE@CuAlO_2_ (GN = 2.0) electrode toward CBZ sensor in phosphate buffer solution (Ph = 7) at the rotation speed = 800 rpm for 2000 s, and electrode potential = 0.75 V. (C) Cyclic stability of GCE@CuAlO_2_ (GN = 2.0) electrode toward CBZ sensor in phosphate buffer solution (Ph = 7) in the presence of 50 μM of CBZ.

Operational stability was probed by continuous amperometry in 50 μM CBZ (0.05 M PBS, pH 7.0) at room temperature for 3000 s (Figure 13B). The oxidation current declined by approximately 2% during the first few hundred seconds and then recovered, stabilizing at 98% of its initial value for the remainder of the test. This minimal signal loss attests to the electrode’s exceptional resistance to fouling and its suitability for prolonged, real-time monitoring. Durability under repeated potential cycling was evaluated by 50 consecutive CV scans in PBS containing 100 μM CBZ (Figure 13C). After the 50th cycle, the anodic-peak current displayed only a 5% decrease relative to the first scan. The corresponding relative standard deviation (RSD) of 3.12% confirms highly reproducible performance and robust structural integrity of the CuAlO_2_ coating. Collectively, these results demonstrate that GCE@CuAlO_2_ (GN = 2.0) exhibits outstanding selectivity, operational longevity, and cyclic durability, underscoring its practicality for on-site, interference-free detection of trace carbendazim in real-world samples.

### 4.12 Repeatability, Reproducibility and Storage Stability Studies of GCE@CuAlO_2_ (GN = 2.0) Electrode toward the Detection of CBZ

The intrinsic repeatability of the GCE@CuAlO_2_ (GN = 2.0) electrode was assessed by cyclic voltammetry in N_2_-purged PBS (pH 7.0) containing 100 μM carbendazim at a scan rate of 150 mVs^–1^. As shown in Figure 14A, five successive scans performed with the same electrode produced nearly identical peak currents, yielding a relative standard deviation (RSD) of 2.16%, which confirms excellent repeatability. Reproducibility was evaluated with five independently prepared GCE@CuAlO_2_ (GN = 2.0) electrodes under identical conditions. The anodic peak currents varied by an RSD of only 2.98% (Figure 14B), demonstrating highly consistent fabrication and response across different sensor units. Storage stability was monitored by recording the oxidation peak of 20 μM CBZ once daily over a period of 15 days, with the sensor stored in pH 7 PBS at 20 °C when not in use (Figure 14C). In 15 days, the peak current retained 90% of its initial value, indicating minimal loss of activity and underscoring the electrodes’ long-term storage stability. Collectively, these metrics confirm that the GCE@CuAlO_2_ (GN = 2.0) sensor consistently delivers dependable performance across repeated measurements, multiple electrodes, and extended storage periods.

**Figure 14:**
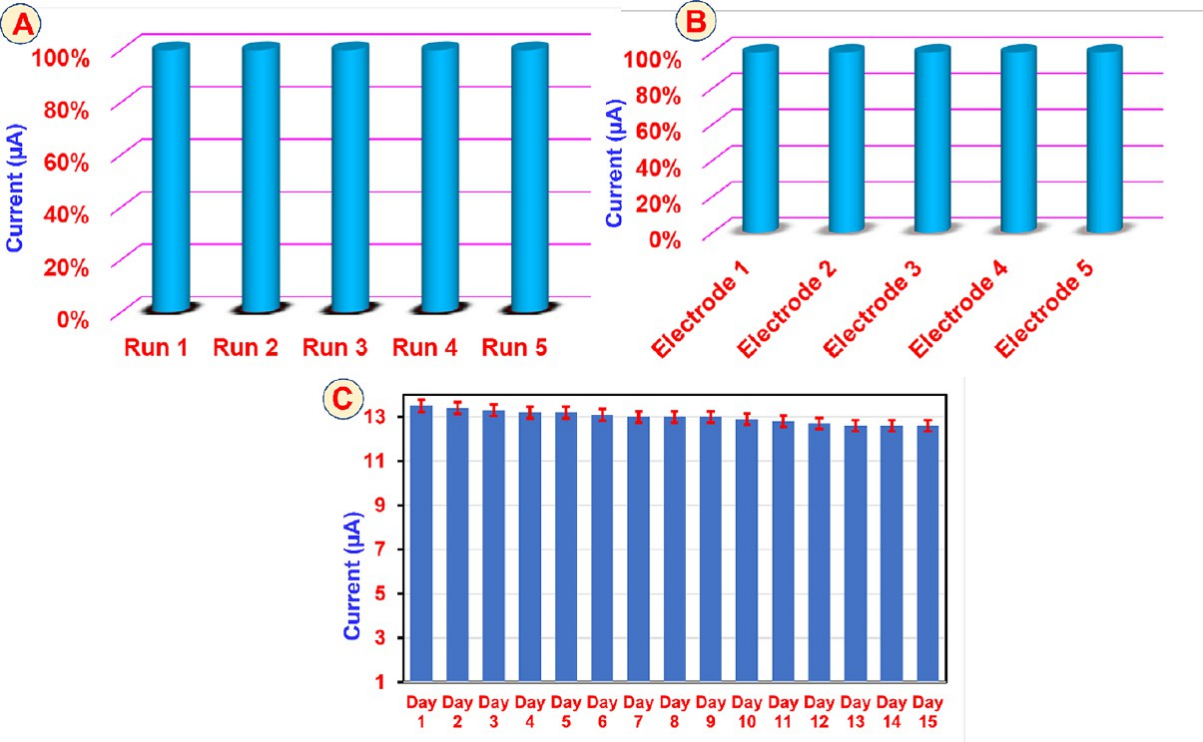
(A) Repeatability, (B) reproducibility, and (C) storage stability studies of the GCE@CuAlO_2_ (GN = 2.0) electrode by the addition of 20 μM of CBZ at 0.05 V s^–1^ in N_2_-immersed PBS.

### 4.13 Real Sample Studies of GCE@CuAlO_2_ (GN = 2.0) Electrode toward the Detection of CBZ

To validate the practical applicability, the sensor was tested on three representative food matrices (A) rice, (B) orange, and (C) cabbage purchased from a local morning market in Taipei, Taiwan. Additionally, water samples were collected from various sources, including (D) tap water, (E) river water (Tamsui River https://maps.app.goo.gl/Wz6f3QTECnoC6t156), and (F) industrial wastewater, in Taipei, Taiwan. Homogenized samples were filtered through 0.45 μm membranes and diluted with pH 7.0 PBS. Known amounts of CBZ were spiked into each matrix, and recoveries were determined using the standard addition method under optimized electrochemical conditions. Recovery experiments were performed at three spiking concentrations (*n* = 3 for each) in different food and water matrices. Each concentration level was tested in triplicate (*n* = 3) to ensure reproducibility. The cyclic-voltammetric responses for the spiked extracts are displayed in Figure 15A–F. Average recoveries of 89.6% (rice), 94.3% (orange), 96.8% (cabbage), 94.4% (Tap water), 97.3% (River water), and 98.2% (Industrial wastewater) were obtained, attesting to the high analytical accuracy of the GCE@CuAlO_2_ (GN = 2.0) electrode in complex, real-world samples. These results confirm that the sensor’s suitability for on-site monitoring of CBZ residues in diverse agricultural products and different water. Electrochemical impedance spectroscopy (EIS) was employed to investigate the interfacial properties of the modified electrode, including polarization resistance (*R*_p_) and the electrical conductivity of the modified surface.

**Figure 15:**
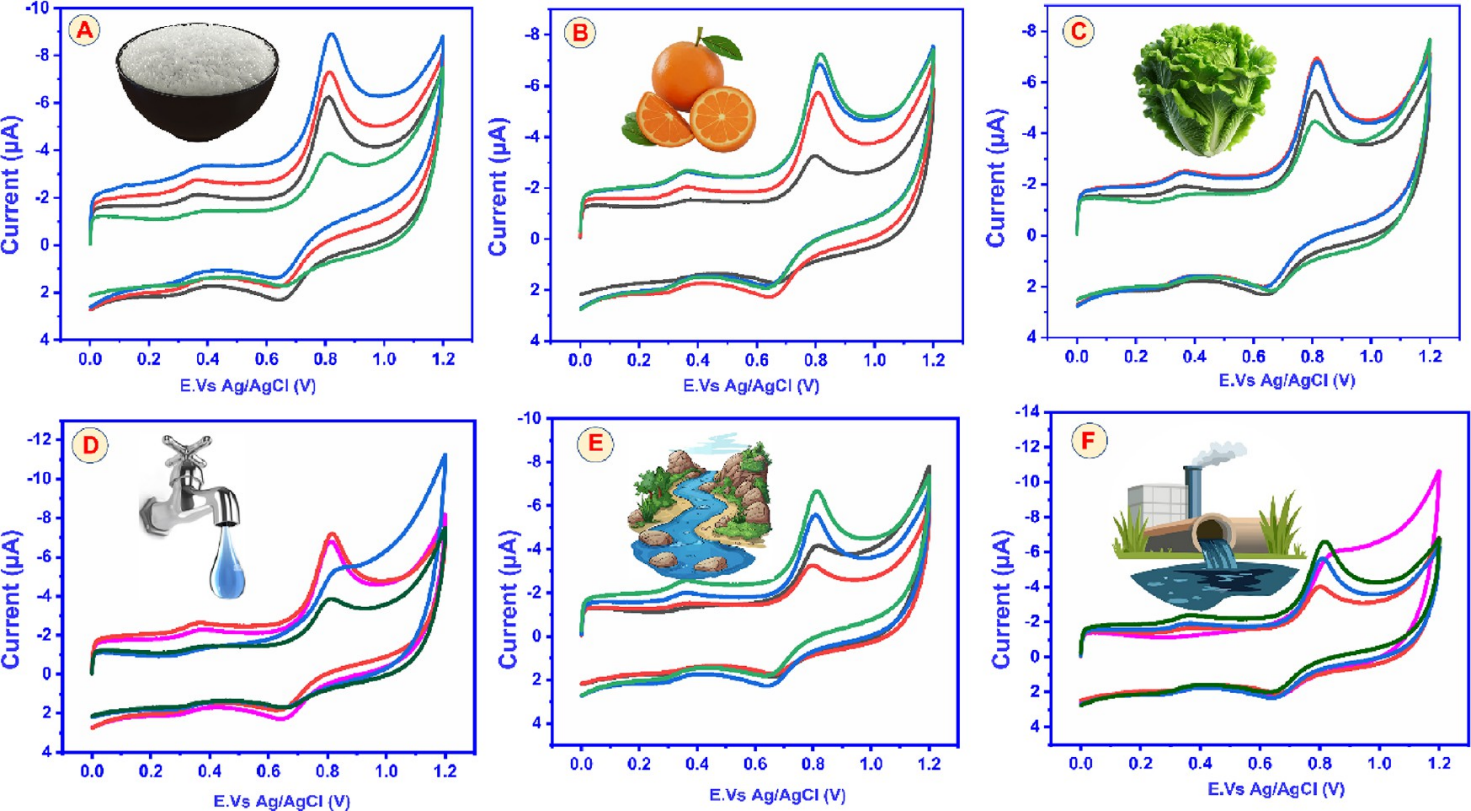
Electrochemical sensing of CBZ with the GCE@CuAlO_2_ (GN = 2.0) electrode in real samples: (A) rice, (B) orange, (C) cabbage, (D) tap water, (E) river water, and (F) industrial wastewater.

In addition to electrochemical testing, CBZ residues in rice, orange, and cabbage samples were quantified using high-performance liquid chromatography (HPLC) for reference purposes. Table 3 summarizes the spiked concentrations, measured recoveries, and comparative results obtained by both techniques. The recovery values achieved with the GCE@CuAlO_2_ (GN = 2.0) electrode are in close agreement with those determined by the conventional HPLC method, confirming excellent concordance between the two approaches. Specifically, the electrochemical and chromatographic recoveries for all three food matrices are virtually identical and fall within the accepted range for trace-level CBZ analysis. These findings demonstrate that the proposed sensor not only delivers rapid, on-site measurements but also matches the accuracy and reliability of laboratory-based HPLC. Consequently, the GCE@CuAlO_2_ (GN = 2.0) electrode provides a robust, accurate, and practical platform for the real-time quantification of CBZ residues in complex food samples.

**Table 3:** Real Sample Analysis of CBZ in Rice, Orange, Cabbage, Tap Water, River Water, and Industrial Wastewater Samples by Using Electrochemical and HPLC Methods

samples	added (μM)	found (μM)	recovery (%)	RSD (%)	HPLC	recovery (%)
rice	10	9.3 ± 0.12	93	3.2	9.0 ± 0.11	90
	20	19.2 ± 0.13	96	3.1	18.7 ± 0.15	93.5
	30	29.5 ± 0.12	98.3	3.5	25.6 ± 0.09	85.3
orange	10	9.7 ± 0.17	97	2.8	9.1 ± 0.12	91
	20	19.8 ± 0.22	99	3.2	19.3 ± 0.23	96.5
	30	29.6 ± 0.23	98.6	2.7	28.7 ± 0.22	95.6
cabbage	10	9.8 ± 0.19	98	3.5	9.5 ± 0.22	95
	20	19.1 ± 0.21	95.5	3.2	19.6 ± 0.27	98
	30	29.0 ± 0.15	96.6	3.7	29.3 ± 0.17	97.6
tap water	10	9.0 ± 0.17	90	2.8	9.1 ± 0.23	91
	20	19.3 ± 0.31	96.5	3.3	19.0 ± 0.35	95
	30	29.5 ± 0.25	98.3	3.1	29.2 ± 0.45	97.3
river water	10	9.6 ± 0.11	96	2.9	9.8 ± 0.21	98
	20	19.3 ± 0.22	96.5	3.8	19.1 ± 0.17	95.5
	30	29.0 ± 0.35	96.6	3.2	29.7 ± 0.18	99
industry water	10	9.4 ± 0.18	94	3.0	9.8 ± 0.24	98
	20	19.2 ± 0.25	96	2.5	19.5 ± 0.35	97.5
	30	29.5 ± 0.36	98.3	2.3	29.8 ± 0.28	99.3

## 5 Computational Studies

The geometry and electronic structure of carbendazim were first optimized with Gaussian 09 ^59^ at the B3LYP/6–31G­(d,p) level. To explore surface interactions, periodic calculations were performed with Quantum ESPRESSO.^58–61^ .Geometry optimizations were carried out for bulk CuAlO_2_ (001) slab and for carbendazim adsorbed on bulk CuAlO_2_, Al-terminated and Cu terminated CuAlO_2_ slabs following the procedure reported,^62^ we employed the GGA + U formalism with U_eff_ = 8 eV to describe the strongly correlated Cu 3d orbitals in CuAlO_2_. Exchange–correlation effects were treated with the Perdew–Burke–Ernzerhof (PBE) functional within the generalized-gradient approximation (GGA). A plane-wave kinetic-energy cutoff of 50 Ry and a charge-density cutoff of 500 Ry were used.^63,64^ Self-consistent-field (SCF) cycles converged to 1 × 10^–8^ eV. In contrast, geometry optimizations were converged to 10^–4^ Ry in total energy and 10^–3^ Ry Bohr^–1^ in residual forces, using the Broyden–Fletcher–Goldfarb–Shanno (BFGS) algorithm.^65–67^ All supercells contained a 20 Å vacuum gap along the z direction to eliminate spurious slab–slab interactions. Brillouin-zone sampling employed the Monkhorst–Pack mesh:^68^ a gamma point (1 × 1 × 1) grid for geometry relaxations adequate for the large surface models, and a denser 2 × 2 × 1 grid for final SCF calculations to refine total energies and capture adsorbate–surface interactions. Kresse represented ionic cores–Joubert PAW (kjpaw) pseudopotentials were used to describe interactions between ions and electrons.^69,70^ Long-range dispersion was included via Grimme’s DFT-D3 correction with Becke–Johnson damping,^71–74^ ensuring accurate adsorption energetics. Optimized structures were inspected with XCrySDen^75,76^ and CrysX-3D Viewer.^77^ Adsorption energies were computed to quantify the stability of carbendazim on both slab terminations, and the resulting geometries were analyzed for key binding motifs. Noncovalent interaction (NCI) analysis, performed using Multiwfn,^78^ further elucidated the nature of adsorbate–surface interactions, distinguishing between hydrogen bonding, π–π stacking, and van der Waals contributions. These comprehensive calculations furnish detailed insight into the adsorption characteristics and stability of the CuAlO_2_–carbendazim systems, complementing the experimental electrochemical findings.

### 5.1 Quantum Chemical Calculations

The Carbendazim molecule has been optimized, revealing a planar configuration composed of carbon (C), hydrogen (H), nitrogen (N), and oxygen (O) atoms, as depicted in Figure 16a. To gain deeper insight into its electronic distribution and potential reactive sites, the Molecular Electrostatic Potential (ESP) map has been generated. This map effectively illustrates regions of varying electron density, providing a clear representation of the molecule’s electronic characteristics and reactive behavior.^79,80^ In the ESP map, red regions indicate zones of highest electron density, typically associated with electron-rich sites. In contrast, dark blue regions correspond to areas of low electron density, indicative of electropositive character. Additionally, light blue areas suggest relatively higher electron density compared to dark blue zones, whereas green regions signify neutral electrostatic potential. As shown in Figure 16a, red zones have been observed over the N and O atoms, signifying them as electron-rich sites. The yellow region over the phenyl ring suggests moderate electron density, while the NH group is enveloped in dark blue, indicating its highly electropositive nature. These findings suggest that the molecule possesses diverse electron density zones that are likely to participate in surface adsorption interactions, especially with metal-oxide substrates such as CuAlO_2_.

**Figure 16:**
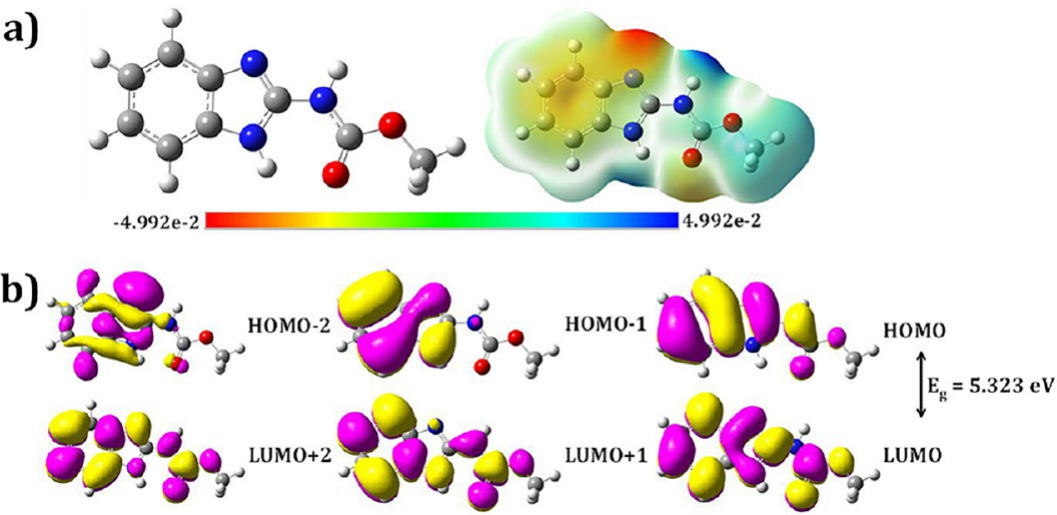
(a) Optimized geometry and ESP (b) FMOs (HOMO–2 to LUMO+2) of the carbendazim molecule obtained at B3LYP/6–31G (d,p) level.

To further understand the molecule’s reactivity and electronic stability, Frontier Molecular Orbital (FMO) analysis has been conducted. The visualizations of molecular orbitals ranging from HOMO–2 to LUMO+2 have been presented in Figure 16b. These FMOs provide crucial information regarding potential charge transfer mechanisms within the molecule. The HOMO and LUMO orbitals are delocalized across the entire molecular structure. In contrast, transitions between HOMO–1 and LUMO+1 indicate charge migration primarily from the octahedron-benzimidazole moiety to other parts of the molecule, suggestive of π–π* transition characteristics. The ESP map and FMO analysis collectively reveal that significant electron density is localized over the lone pairs of the N and O atoms, the NH group, and the OCH_3_ moiety, as observed in the HOMO orbital. These electron-rich regions are likely to interact with the unoccupied *d*-orbitals of the CuAlO_2_ surface through electron donation, underscoring their potential role in the adsorption process.^81^

The adsorption of the Carbendazim molecule onto the CuAlO_2_ slab has been initiated by first evaluating the possible interaction sites within the CuAlO_2_ bulk structure, as depicted in Figure 17a–c. Based on a previous study,^82^ the bulk CuAlO_2_ has been modeled using a delafossite-type lattice structure with R $\overset{-}{3}$ m symmetry, which belongs to the trigonal crystal system. For computational convenience and to enable accurate prediction of surface–molecule interactions, this structure has been represented in the hexagonal setting. To identify potential adsorption sites, the CuAlO_2_ slab model is constructed by cleaving the optimized bulk structure along its (001) surface, as shown in Figure 17a–c. The atomic layer arrangement within the CuAlO_2_ slab has been systematically analyzed. The topmost layer consists of oxygen atoms, followed by a second layer comprising both aluminum and oxygen atoms, and a third layer composed of copper atoms. The layered atomic structure, consisting of oxygen (O) atoms at the surface, followed sequentially by aluminum (Al) and copper (Cu), facilitated the creation of slab models with either Al- or Cu-terminated surfaces. This configuration enabled a systematic exploration of how surface termination influences the adsorption characteristics of the Carbendazim molecule. To investigate the adsorption behavior of the Carbendazim molecule over the CuAlO_2_ slab, the adsorption energy (*E*_ads_) has been calculated by using the following eq 13:

$$E_{\mathrm{ads}}=E_{\mathrm{Bulk}\;\mathrm{CuAlO}2/\mathrm{CuAlO}2-\mathrm{Al}-\mathrm{terminated}\;\mathrm{surface}/\mathrm{Cu}-\mathrm{terminated}\;\mathrm{surface}-\mathrm{carbendazim}\;\mathrm{molecule}}-(E_{\mathrm{Bulk}\;\mathrm{CuAlO}2/\mathrm{CuAlO}2-\mathrm{Al}-\mathrm{terminated}\;\mathrm{surface}/\mathrm{Cu}-\mathrm{terminated}\;\mathrm{surface}}+E_{\mathrm{carbendazim}\;\mathrm{molecule}})$$ 13

**Figure 17:**
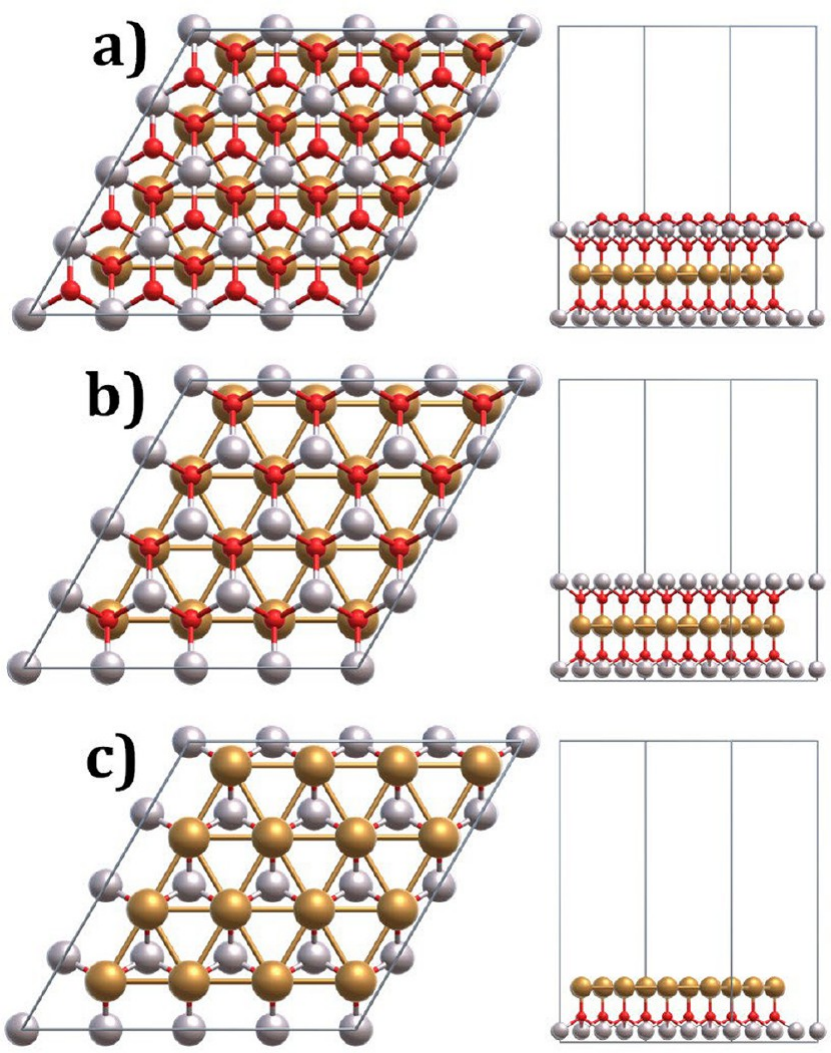
Top and side view of optimized structure of CuAlO_2_ (001): (a) bulk CuAlO_2_ structure, (b) slab model cleaved to expose Al-terminated surface, and (c) slab model cleaved to expose Cu-terminated surface.

Where EBulk CuAlO_2_/CuAlO_2_-Al-terminated surface/Cu-terminated surface – carbendazim molecule, *E*_Bulk_ CuAlO_2_/CuAlO_2_-Al-terminated surface/Cu-terminated surface, and *E*_carbendazim molecule_ are the total energy of the system containing bulk CuAlO_2_/CuAlO_2_-Al-terminated surface/Cu-terminated surface with carbendazim molecule, bulk CuAlO_2_/CuAlO_2_-Al-terminated surface/Cu-terminated surface, and carbendazim molecule, respectively. Initially, the Carbendazim molecule was placed over the top layer of the CuAlO_2_ slab, which consists predominantly of oxygen atoms, as shown in Figure 18a–c. Geometry optimization has been carried out for this configuration, resulting in an *E*_ads_ of −2.897 eV as shown in Figure 18a–c. As reported in the literature, the more negative the *E*_ads_ value, the stronger the adsorption of the adsorbate molecule onto the adsorbent.^82^ The optimized structure has revealed a hydrogen bond interaction between the N–H group of the Carbendazim molecule and the surface O atom (N–H---O), with a short interaction distance of 1.706 Å. This short distance suggests strong adsorption efficacy, as shorter interactions typically correspond to stronger binding.^82^ To further validate the nature of the interaction, an NCI analysis has been performed. The NCI plot (Figures 19a–c) shows the presence of both hydrogen bonding and van der Waals (vdW) interactions. Specifically, regions with negative values of sign­(λ_2_)­ρ­(*r*) < 0 indicate strong, attractive interactions, such as hydrogen bonding, while regions near zero correspond to weaker van der Waals forces. Following this, the adsorption was examined on the second atomic layer, which was identified as Al-terminated. The adsorption of Carbendazim on this Al-terminated surface yielded an *E*_ads_ value of −1.919 eV, which is 0.978 eV less negative than the value obtained for the O-terminated top layer. In this case, the shortest interaction distance between the Al atom and the N atom (bearing a lone pair) from the Carbendazim molecule (C–N---Al) has been found to be 3.556 Å, significantly longer (∼1.85 Å more) than the corresponding N–H···O interaction in the O-terminated configuration. Despite the weaker interaction, the NCI plot (Figure 19b) still reveals slight attractive interactions, along with van der Waals (vdW) forces, suggesting that weak adsorption has occurred on the Al-terminated surface. The investigation has been further extended by cleaving the CuAlO_2_ slab to expose a Cu-terminated surface.

**Figure 18:**
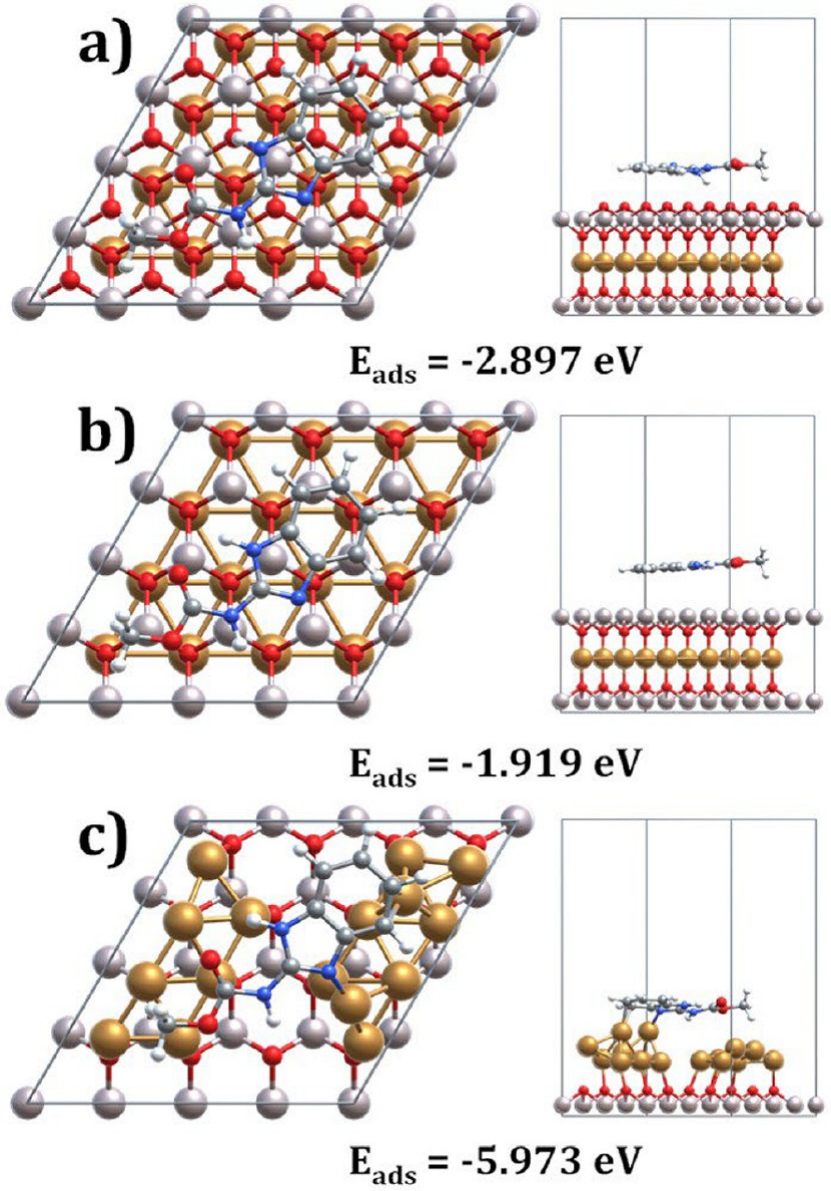
Top and side views of carbendazim adsorbed onto (a) bulk CuAlO_2_ structure, (b) Al-terminated CuAlO_2_ slab surface, and (c) Cu-terminated CuAlO_2_ slab surface.

**Figure 19:**
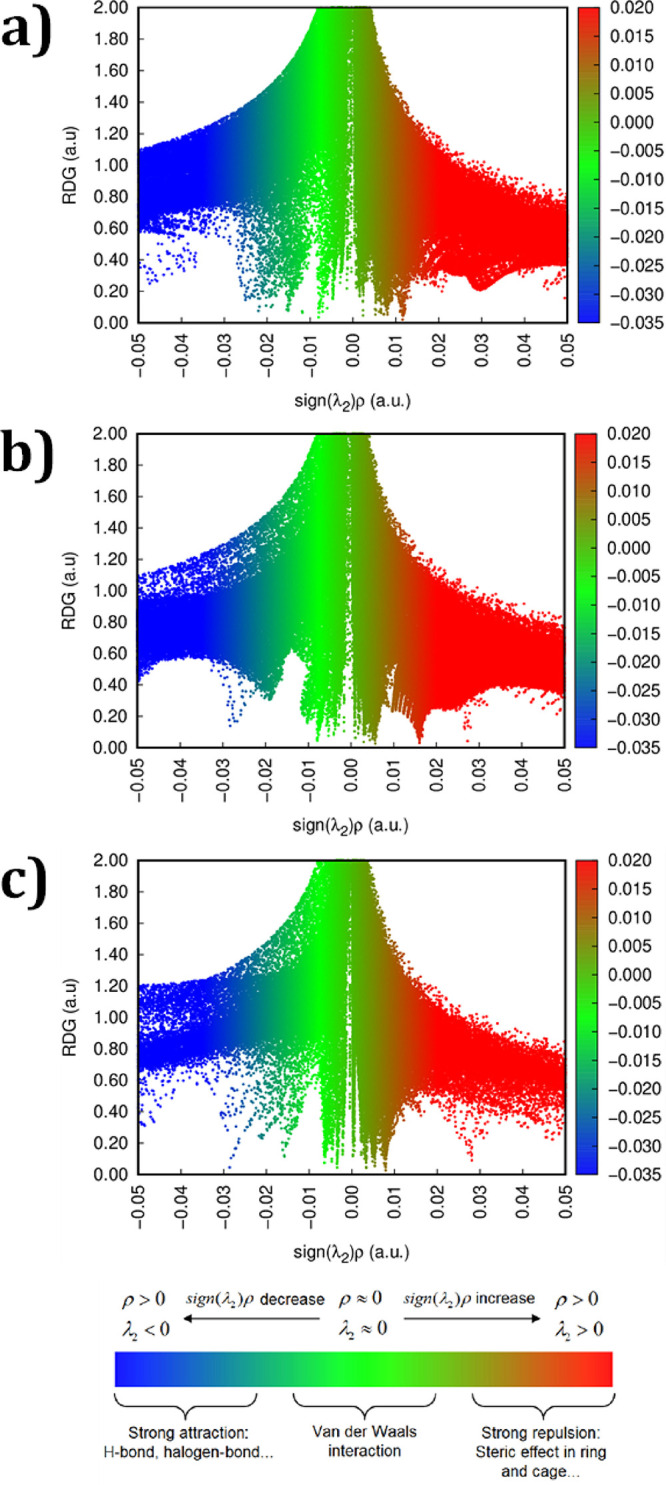
NCI-scatter plot of carbendazim adsorbed onto (a) bulk CuAlO_2_ structure, (b) Al-terminated CuAlO_2_ slab surface, and (c) Cu-terminated CuAlO_2_ slab surface.

As anticipated, the N atom in the Carbendazim molecule, with its lone electron pair, has shown a strong interaction with the exposed Cu atom. This has been supported by the previously discussed ESP map, where the lone pair region of the N appeared electron-rich (red region). The resulting interaction distance has been calculated to be 1.955 Å, indicating a much stronger interaction compared to the Al-terminated case. The corresponding *E*_ads_ have been determined to be −5.973 eV, which is 4.054 eV more negative than that of the Al-terminated surface, highlighting the significantly enhanced adsorption on the Cu-terminated slab. Additionally, the NCI plot (Figure 19c) for this configuration shows prominent attractive regions and van der Waals interactions, further confirming the strong binding between the Carbendazim molecule and the Cu surface. Overall, these findings have confirmed that the Carbendazim molecule preferentially adsorbs on the Cu-terminated surface of the CuAlO_2_ slab, as evidenced by the more negative *E*_*ads*_ value. A more negative adsorption energy indicates a more favorable interaction, thus confirming the Cu-terminated surface as the most energetically favorable site for Carbendazim adsorption.

## 6 Conclusions

In this study, CuAlO_2_ nanopowder was successfully synthesized using a highly efficient self-combustion glycine nitrate process (GNP). The structural, morphological, and compositional features of the synthesized CuAlO_2_ were thoroughly characterized using various techniques, including XRD, FE-SEM, EDX, HR-TEM, FT-IR, and XPS. These analyses confirmed the formation of a delafossite-type CuAlO_2_ structure with desirable microstructural and physicochemical properties. Electrochemical investigations were performed using cyclic voltammetry (CV) and amperometric (*i*–*t*) techniques. A simple, disposable, and cost-effective GCE@CuAlO_2_ (GN = 2.0) modified electrode was fabricated and utilized for the sensitive detection of CBZ. Among the various formulations tested GCE, GCE@CuAlO_2_ (GN = 1.0), GCE@CuAlO_2_ (GN = 1.3), and GCE@CuAlO_2_ (GN = 1.5). The GCE@CuAlO_2_ (GN = 2.0) electrode exhibited the most pronounced redox activity toward CBZ detection. The optimized GCE@CuAlO_2_ (GN = 2.0) electrode demonstrated remarkable analytical performance, including an ultralow detection limit of 1 nM, high sensitivity of 1.44 μAμM^–1^cm^–2^, and a wide linear detection range from 0.01 to 800 μM. Its superior electrocatalytic activity was attributed to the synergistic effect of the CuAlO_2_ complex, high surface area, enhanced electronic conductivity, and the abundance of electroactive sites. Furthermore, the developed electrochemical sensor demonstrated excellent applicability in real samples. It achieved high average recovery rates of 89.6, 94.3, 96.8, 97.3, 99.0, and 99.3% for rice, orange, cabbage, tap water, river water, and industrial wastewater samples, respectively. These results were closely comparable to those obtained using the conventional HPLC method, validating the accuracy and reliability of the proposed sensor system. The GCE@CuAlO_2_ (GN = 2.0) electrode presents a promising, cost-effective, and sensitive platform for the real-time electrochemical detection of trace levels of CBZ. Using DFT calculations, the electronic properties of the CBZ molecule have been investigated to evaluate its adsorption potential on the CuAlO_2_ slab. Strategic adsorption studies have been conducted on the bulk CuAlO_2_ structure, the Al-terminated surface, and the Cu-terminated surface. Among these, the Cu-terminated CuAlO_2_ surface has exhibited the strongest interaction, as further supported by the NCI scatter plot analysis, which has provided insight into the nature of noncovalent interactions. The highest *E*_ads_ value recorded is −5.973 eV. Its simplicity, reproducibility, and strong anti-interference capability make it an ideal candidate for routine monitoring of pesticide residues in food, pharmaceutical, and environmental samples.
